# Deterministic programming of human pluripotent stem cells into microglia facilitates studying their role in health and disease

**DOI:** 10.1073/pnas.2123476119

**Published:** 2022-10-17

**Authors:** Anna M. Speicher, Lisanne Korn, Júlia Csatári, Laura Gonzalez-Cano, Michael Heming, Christian Thomas, Christina B. Schroeter, David Schafflick, Xiaolin Li, Lukas Gola, Alexander Engler, Thilo Kaehne, Ludovic Vallier, Sven G. Meuth, Gerd Meyer zu Hörste, Stjepana Kovac, Heinz Wiendl, Hans R. Schöler, Matthias Pawlowski

**Affiliations:** ^a^Department of Neurology with Institute of Translational Neurology, Universitätsklinikum Münster, 48149 Münster, Germany;; ^b^Department of Cell and Developmental Biology, Max Planck Institute for Molecular Biomedicine, 48149 Münster, Germany;; ^c^Institute of Neuropathology, Universitätsklinikum Münster, 48149 Münster, Germany;; ^d^Institute of Experimental Internal Medicine, Otto von Guericke Universität, 39106 Magdeburg, Germany;; ^e^Cambridge Stem Cell Institute, University of Cambridge, Cambridge CB2 1TN, United Kingdom;; ^f^Berlin Institute of Health-Center for Regenerative Therapies, Berlin Institute of Health, Charité-Universitätsmedizin Berlin, 13353 Berlin, Germany;; ^g^Medical Faculty, University of Münster, 48149 Münster, Germany

**Keywords:** stem cells, microglia, reprogramming, brain organoids, neurodegeneration

## Abstract

Microglia, the resident immune cells of the central nervous system (CNS), are derived from yolk-sac macrophages that populate the developing CNS during early embryonic development. Once established, the microglia population is self-maintained throughout life by local proliferation. As a scalable source of microglia-like cells (MGLs), we here present a forward programming protocol for their generation from human pluripotent stem cells (hPSCs). The transient overexpression of PU.1 and C/EBPβ in hPSCs led to a homogenous population of mature microglia within 16 d. MGLs met microglia characteristics on a morphological, transcriptional, and functional level. MGLs facilitated the investigation of a human tauopathy model in cortical neuron–microglia cocultures, revealing a secondary dystrophic microglia phenotype. Single-cell RNA sequencing of microglia integrated into hPSC-derived cortical brain organoids demonstrated a shift of microglia signatures toward a more-developmental in vivo–like phenotype, inducing intercellular interactions promoting neurogenesis and arborization. Taken together, our microglia forward programming platform represents a tool for both reductionist studies in monocultures and complex coculture systems, including 3D brain organoids for the study of cellular interactions in healthy or diseased environments.

## Significance

We here present a method for the manufacture of pure bulk quantities of microglia from human pluripotent stem cells (hPSCs) at unprecedented efficiency. We provide transcriptional, proteomic, and functional analysis of the microglia in two-dimensional (2D) cultures and single cell–resolution transcriptional profiling in 3D cortical organoids. This versatile technology of hPSC-derived microglia will improve in vitro models of the human brain and neurological disease. The platform will also facilitate biomedical research, including compound screening, drug discovery, and transplantation studies. We further demonstrate differential secondary *MAPT* genotype–dependent, microglial disease–associated phenotypes when microglia were placed in coculture with tau mutant cortical neurons. These findings provide evidence for mutation-dependent, differential pathophysiological effects on the immune response in the hereditary tauopathies.

Microglia have key roles in central nervous system (CNS) development and in maintaining brain homeostasis (1). Upon homeostatic disturbance, microglia react with the secretion of cytokines, increased production of reactive oxygen species (ROS), phagocytosis, and antigen presentation, thus enabling immune responses (1–4). Microglia are implicated in the onset and progression of many CNS diseases. Hence, there is an urgent need for robust cell-culture models to facilitate the study of microglia and their role in disease. Due to several interspecific differences between mice and men, mouse models fail to accurately represent human conditions. For example, mice completely lack orthologs of several risk genes associated with neurodegenerative diseases (e.g., *CD33* and *CR1*), and for others, homology between mouse and human proteins is low (5, 6). Moreover, whole transcriptome–sequencing studies reported significant differences in the expression of inflammatory cytokines, complement factors, and other genes related to neuroinflammation and neurodegeneration between human and rodent cells (7, 8). Purified microglia from postmortem human brain tissue are difficult to obtain and lack the scalability required for many biotechnological and medical applications. Since late 2016, several protocols for the generation of microglia-like cells (MGLs) from human pluripotent stem cells (hPSCs) have been reported, thus enabling the scalable production of human MGLs from a renewable source. However, all available protocols exhibit major drawbacks, including long culture durations (up to 74 d) (9), requirement for mechanical manipulation steps or enrichment of certain cell populations by magnetic- or fluorescent-activated cell sorting (FACS), or the lack of defined culture conditions due to the need for embryoid-body formation or serum-containing media formulations (10). This severely limits reproducibility and scalability. Despite these caveats, studies using hPSC-derived MGLs have expanded our understanding of human microglia identity in vitro and in vivo and they have been successfully used to investigate different mutations associated with neurodegenerative diseases (10, 11).

In this study, we present a deterministic forward programming protocol for the generation of MGLs from hPSCs at unprecedented speed (16 d) and efficiency (100%). We provide extensive characterizations to confirm the identity of our hPSC-derived MGLs by protein and whole transcriptome–expression analysis and a variety of functional and metabolic assays. Cocultures with hPSC-derived cortical neurons or cortical organoids complement the protocol to form a versatile toolkit for microglia studies from reductionist microglia monocultures to complex CNS cocultures. Using the two-dimensional (2D) microglia–neuron coculture system, we demonstrated genotype-dependent MGL activation, leading to deleterious ROS production and imbalance of the redox system in tauopathies. 3D cocultures with brain organoids were implemented to show that cell–cell interactions in a more-complex environment alter the single-cell MGL transcriptome.

## Results

### Forward Programming of Human Induced Pluripotent Stem Cells (hiPSCs) into MGLs by Overexpression of PU.1 and C/EBPβ.

**Fig. 1.**
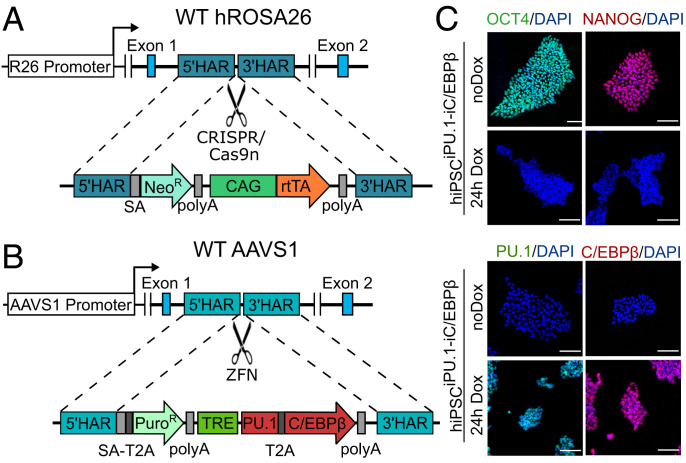
Gene-targeting strategy of the dual GSH-based inducible overexpression system. (*A*) The hROSA26 locus was targeted using CRISPR/Cas9n for insertion of the constitutive CAG-rtTA expression cassette. (*B*) The AAVS1 locus was targeted using ZFNs for insertion of the inducible biscistronic reprogramming factor cassette for timed expression of the TFs PU.1 and C/EBPβ. (*C*) Immunocytochemistry (ICC) for OCT4 (green) and NANOG (red) and PU.1 (green) and C/EBPβ (red) under control conditions and after 24 h of induction with Dox. Abbreviations: 5′-HAR/3′-HAR: upstream/downstream homology arm; AAVS1: AAVS1 locus (19q13.42); CAG: cytomegalovirus early enhancer, chicken β-actin, and rabbit β-globin hybrid promoter; Neo^R^: neomycin resistance; Puro^R^: puromycin resistance; R26: hROSA26 locus (3p25.3); rtTA: reverse tetracycline transactivator; SA: splice acceptor; T2A: T2A peptide (ribosomal skipping signal); TRE: Tet-responsive element; WT: wild type; ZFN: zinc finger nuclease. Scale bars = 100 µm.

We first sought to develop a deterministic forward programming protocol based on forced expression of the transcription factors (TFs) PU.1 (encoded by *SPI1*) and C/EBPβ (encoded by *CEBPB*) in hiPSCs. PU.1 plays a nonredundant role in the development of the myeloid cell lineage (12, 13), and lineage-tracing studies of microglia have revealed high expression levels of PU.1 and C/EBPβ throughout embryonic microglia development (8). The combination of these two TFs induces some myeloid cell-surface markers in a mouse embryonic fibroblast line (14). We adapted our previously reported dual genomic safe harbor (GSH) targeting of the Tet-ON system for inducible, timed, and silencing-resistant transgene expression in hiPSCs (15, 16) (Fig. 1 *A* and *B* and *SI Appendix*, Fig. S1). After 24 h of doxycycline (Dox) treatment, we detected strong and homogenous PU.1 and C/EBPβ transgene expression in all cells while expression of the pluripotency markers OCT4 and NANOG was abrogated (Fig. 1*C*).

**Fig. 2.**
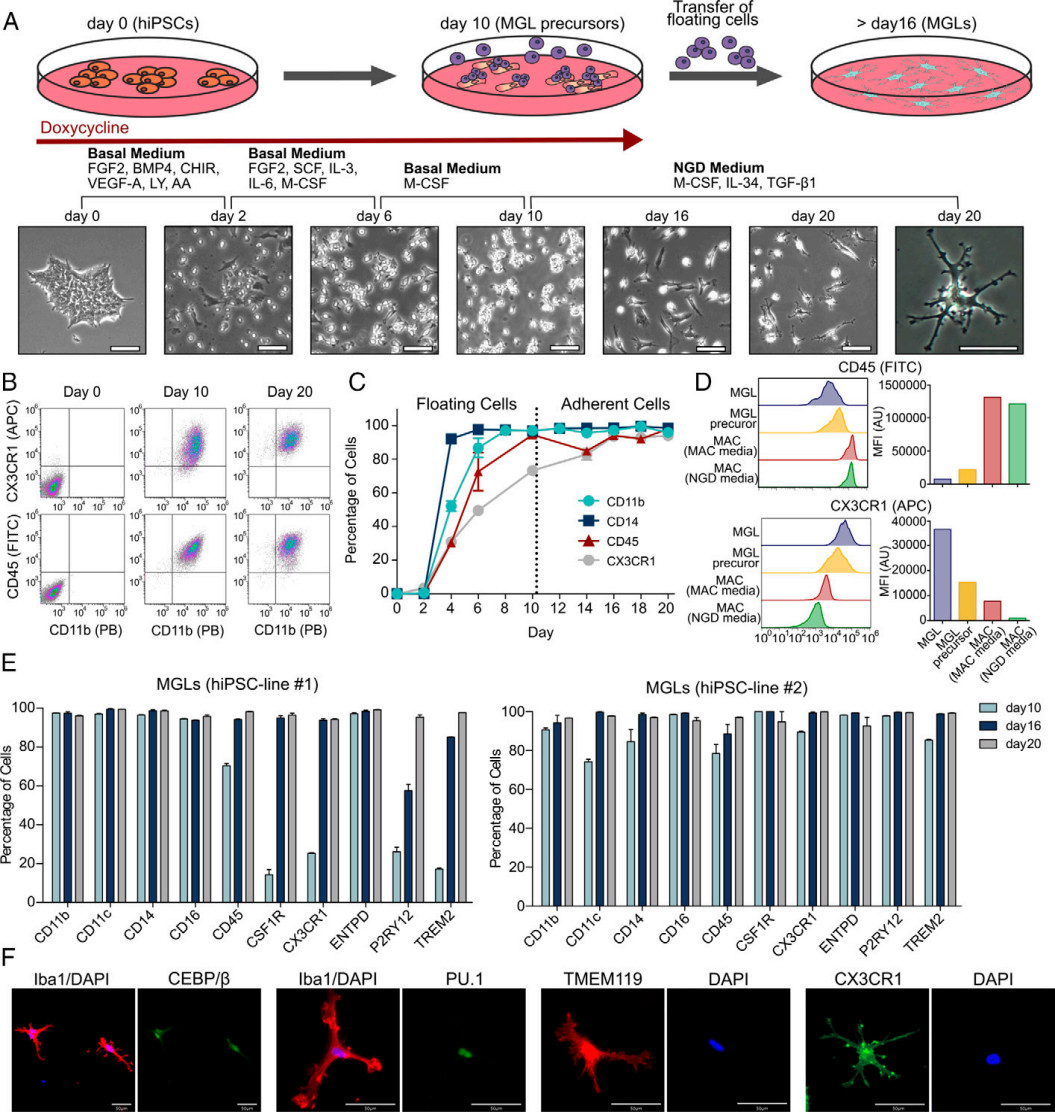
MGL forward programming protocol and MGL characterization. (*A*) Schematic workflow of MGL generation: hiPSCs are induced with Dox in a basal medium containing small molecules and cytokines promoting posterior primitive streak formation and primitive hematopoiesis. The medium is changed every other day and is supplemented with the indicated cytokines for promoting primitive hematopoiesis and primitive yolk-sac macrophage maturation. After 10 d, detached MGL precursors are collected and replated onto PLL-coated culture dishes in the final NGD medium without Dox. Phase-contrast images illustrate the morphological changes throughout the protocol. (*B*) Flow cytometry plots showing the myeloid markers CX3CR1/CD11b and CD14/CD11b on days 0, 10, and 20 of the protocol. (*C*) Time course showing expression levels of myeloid cell-surface markers (CD11b, CD14, CD45, and CX3CR1) from days 0 to 20. (*D*) Comparison of CD45 and CX3CR1 expression levels in MGLs, MGL precursors, and primary macrophages (MACs) cultivated in macrophage or microglia medium. (*E*) Expression of myeloid and microglia cell-surface markers in two independent hiPSC lines on days 10, 16, and 20 of our protocol. (*F*) ICC for IBA1 in costaining with the TFs PU.1 and C/EBPβ and for the microglia surface-marker proteins TMEM119 and CX3CR1. Abbreviations: FGF: fibroblast growth factor; LY: LY294002 hydrochloride; AA: ascorbic acid; FITC: Fluorescein-5-isothiocyanat; APC: Allophycocyanin; PB: Pacific blue.

Recent evidence highlights the importance of the interplay between extracellular signaling and reprogramming factors for efficient programming of the desired target cell type (16–18). Here, we sequentially exposed the Dox-induced cells to cytokines and small molecules mimicking posterior primitive streak development, primitive hematopoiesis, and primitive yolk-sac macrophage maturation, respectively (Fig. 2*A*). However, TF overexpression remains essential and complete omission of Dox did not lead to macrophage or microglia generation as measured by cell surface marker expression (*SI Appendix*, Fig. S2). By days 8–10, all cells detached from the culture dish and expressed common myeloid surface markers (CD11b, CD14, CD45, and CX3CR1; Fig. 2 *B* and *C*). These floating cells (henceforth named MGL precursors) were collected and replated on new culture dishes. At this point, Dox was withdrawn from the media to turn off transgene expression, and PU.1 and C/EBPβ expression was afterward solely driven by the respective endogenous loci (see below). Medium was switched to cerebrospinal fluid–mimicking neuroglial differentiation (NGD) medium (9), supplemented with interleukin (IL)-34, macrophage colony-stimulating factor, and transforming growth factor (TGF)-β1, which have been shown to be involved in development and maintenance of homeostatic microglia (19, 20). A flow cytometry time course of the myeloid surface markers CD11b, CD14, CD45, and CX3CR1 demonstrated robust expression of all four markers by day 16 (Fig. 2*C*). None of these markers are specific to the microglia lineage, but in some cases, their expression levels may be used to distinguish microglia from related macrophage phenotypes. Direct comparison of MGLs and human peripheral blood–derived macrophages (MACs) by flow cytometry demonstrated higher expression levels of CX3CR1 and lower levels of CD45 in MGLs compared with MACs (21, 22). These differences were maintained even when MACs were cultured in NGD medium, confirming that MGLs were CD45^int^/CX3CR1^high^ while MACs in both media were CD45^high^/CX3CR1^int^ (Fig. 2*D*). Extended multicolor flow cytometry on day 10, 16, and 20 demonstrated expression of additional canonical microglia-lineage surface proteins, including the CSF1 receptor CD115 and the purinergic receptor P2Y12 and TREM2 (Fig. 2*E*) (21). These microglia/myeloid markers were expressed by all cells generated from two independent hiPSC lines (Fig. 2*E*). Immunocytochemistry confirmed that all cells expressed the human microglia markers IBA1, CX3CR1, and TMEM119 and the TFs PU.1 and C/EBPβ (Fig. 2*F*). Taken together, the presented protocol allowed the generation of pure MGL populations with homogenous marker protein expression within 16 d. The yield of MGLs is approximately two times the amount of hiPSCs initially seeded.

### MGLs Resemble Primary Microglia and Differ from MACs.

**Fig. 3.**
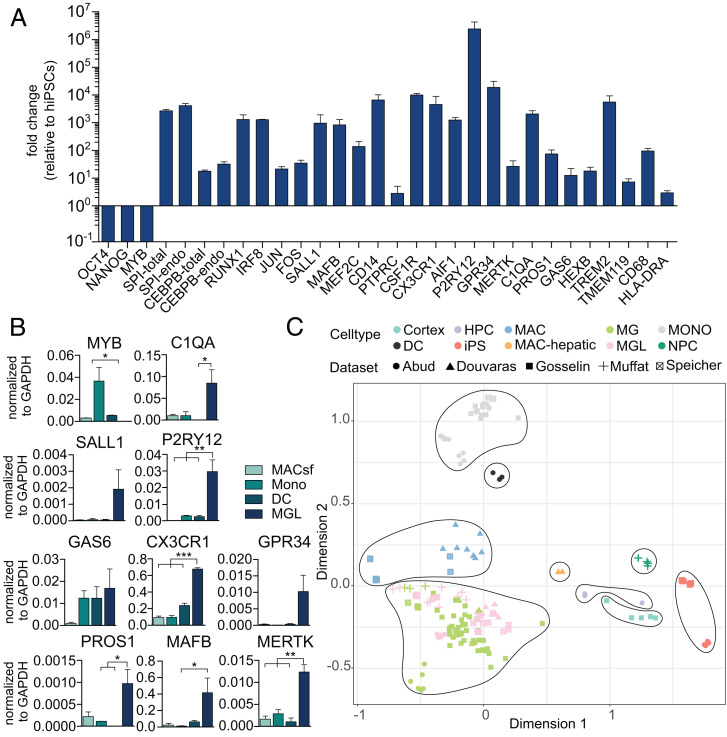
Marker gene expression and transcriptome of MGLs compared with stem cells and other myeloid cell types. (*A*) Real-time qPCR showing gene-expression levels of the pluripotency factors, the MAC TF *MYB*, core microglia network TFs, core microglia signature genes, and typical microglia cell-surface proteins in MGLs. (*B*) Comparison of expression levels of microglia signature genes and MYB in MGLs versus peripheral blood–derived MACs (serum-free), monocytes (Monos), and dendritic cells (DCs). Statistical significance was determined using Bonferroni-corrected one-way ANOVA (*P < 0.05; **P < 0.01; ***P < 0.001). (*C*) Multidimensional scaling plot of bulk RNA sequencing data of our MGLs generated with different media conditions, human postmortem microglia ex vivo, PBMC-isolated monocytes, and PBMC-derived MACs with different media conditions. Moreover, the plot contains RNA-expression profiles of MGLs from three previously published protocols (9, 19, 25), including additional cell types for comparison, and an extensive dataset of primary human microglia in vitro and ex vivo, MACs, and monocytes (8).

Next, we investigated the gene-expression profile of MGLs and benchmarked their expression against hiPSCs, MACs, and primary microglia. Quantitative real-time PCR demonstrated downregulation of pluripotency factors (*OCT4* and *NANOG*), whereas core microglia lineage TFs (*SPI1*, *CEBPB*, *RUNX1*, and *IRF8*), additional developmentally or environmentally controlled microglia TFs (*JUN*, *FOS*, *MAFB*, *MEF2C*, and *SALL1*) (8), microglia signature genes (*GAS6*, *C1QA*, *PROS1*, *HEXB*, *MERTK*, *P2RY12*, and *GPR34*) (20), and other typical microglia markers, including *AIF1* (encoding IBA1) and *TMEM119* (21, 23), were strongly upregulated in MGLs compared with hiPSCs. Importantly, primers detecting either total *SPI1* and *CEBPB* transcripts or only those from the respective endogenous loci revealed equal expression levels, thus indicating complete independence of the microglia phenotype from transgene expression (Fig. 3*A*). Moreover, we did not detect expression of the monocyte/MAC TF *MYB* in MGLs (Fig. 3*A*). Taken together, this expression profile is in line with the transcriptional identity of microglia descending from primitive (PU.1-, C/EBPβ-, RUNX1-, and IRF8-dependent) and not definitive (MYB-dependent) hematopoiesis (24). Moreover, we confirmed higher expression levels of all members of the previously reported microglia signature profile (*P2RY12*, *GAS6*, *MERTK*, *C1QA*, *PROS1*, and *GPR34*), *CX3CR1*, and the key microglia TF *SALL1* in MGLs compared with MACs (serum-free), monocytes, and dendritic cells (DCs) isolated or derived from peripheral blood mononuclear cells (PBMCs), while *MYB* expression was only detected in MACs, monocytes, and DCs (Fig. 3*B*).

To further verify the cellular identity of MGLs and distinguish them from related cell types, we performed bulk RNA sequencing directly comparing MGLs with hiPSCs; primary PBMC-derived MACs in serum-containing, serum-free, or NGD-medium, PBMC-isolated monocytes and DCs; and primary human adult microglia ex vivo. Moreover, we included previously published datasets into our analysis, including hiPSC-derived MGLs generated by three different classical differentiation protocols (9, 19, 25) and an extensive dataset of human primary microglia in vitro and ex vivo (8). Multidimensional scaling analysis of the integrated datasets (dimension 1 versus 2) showed several clusters. All microglia and MGL datasets cluster together, and MACs form a different cluster and monocytes as well. Further clusters that emerged in our analysis are DCs, hepatic MACs, hematopoietic precursor cells, neural progenitor cells, cells isolated from cortical tissue, and hiPSCs (Fig. 3*C*). When removing the strongly contrasting stem-cell populations and cortical cells, all MGLs form a cluster that partially overlaps with primary human microglia in vitro but is distinct from all other myeloid cell types (*SI Appendix*, Fig. S3). Heatmaps illustrate comparison of expression levels of a previously defined large set of genes enriched in primary human microglia ex vivo (8) and demonstrate clustering of MGLs with ex vivo microglia, whereas MACs, monocytes, and hiPSCs form separate clusters (*SI Appendix*, Fig. S4).

Aiming to simplify the presented protocol, we tested media complexity reductions for MGL programming. We omitted single cytokines or cytokine combinations from the programming protocol and analyzed the different culture conditions (*SI Appendix*, Fig. S5*A*) by qPCR and bulk RNA sequencing with primary human microglia and MGLs produced with our standard protocol as surrogate of MGL identity. Consistent with previous protocols (18), we found that extracellular cues known to induce patterning of posterior primitive streak/extraembryonic mesoderm (e.g., BMP-4) (26) were required for successful programming, as omission did not yield viable cell populations (*SI Appendix*, Fig. S5*B*). Likely, these extracellular signals facilitate the context-dependent action of the two reprogramming factors. However, omission of some cues, i.e., either vascular endothelial growth factor (VEGF)-A and the glycogen synthase kinase 3 inhibitor CHIR (day 0–2), or stem cell factor (SCF), IL-3, and IL-6 (d2-6) were dispensable, as indicated by marker gene expression (*SI Appendix*, Fig. S5*C*) and transcriptome sequencing (Fig. 3*C*). Morphological assessment showed that cells generated in the absence of VEGF-A and CHIR (condition 3) had a rounder shape. The same was found for alternatively modulated WNT signaling in combination with omission of SCF, IL-3, and IL-6 (condition 8). Cytokine secretion changed in the absence of SCF, IL-3, and IL-6 insofar that the cells secreted significantly more IL-6 and more IL-12p40 compared with standard conditions. With modulated WNT signaling in combination with omission of SCF, IL-3, and IL-6, the secretion of IL-23 and thymus- and activation-regulated chemokine (TARC) was significantly lower than in standard MGLs. All other cytokines examined were secreted at similar levels (*SI Appendix*, Fig. S5 *B* and *D*). Bulk RNA sequencing revealed no major differences between MGLs produced with our standard protocol versus simplified media conditions (conditions 2, 3, and 7) (Fig. 3*C* and *SI Appendix*, Fig. S3)

### MGLs Functionally and Metabolically Exhibit Core Microglia Features.

**Fig. 4.**
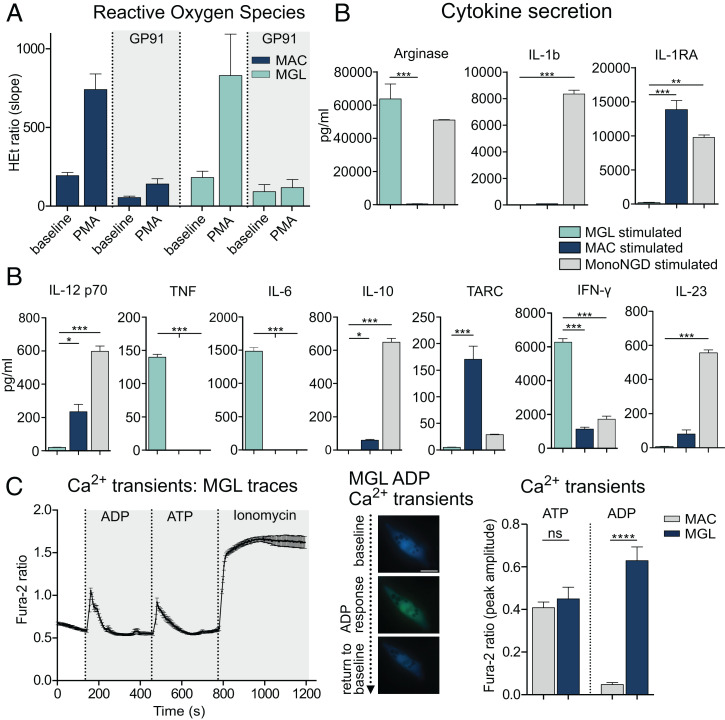
MGLs exert classical myeloid and microglia-specific functions. (*A*) ROS production of MGLs and MACs at baseline and responding to treatment with the NADPH-oxidase activator PMA under standard conditions and after treatment with the selective NOX2 inhibitor GP91-TAT. (*B*) Differentially secreted cytokines in MGLs, MACs, and monocytes in NGD medium (MonoNGD) stimulated with IFN-γ and LPS. (*C*) Representative trace of Ca^2+^ oscillations induced by sequential stimulation of MGLs with ADP, ATP, and ionomycin (*Left*). Upon stimulation with ADP, MGLs respond with an increased fluorescence, indicating Ca^2+^ influx, and a subsequent return to baseline, indicating Ca^2+^ efflux (*Middle*). Quantification of calcium transients in MGLs and MACs evoked by ATP or ADP stimulation (*Right*). Statistical significance was determined using Bonferroni-corrected one-way ANOVA (ns: not significant; *P < 0.05; **P < 0.01; ***P < 0.001).

A key microglia function is phagocytosis of cell debris, pathogens, and other brain homeostasis–disturbing particles. With a phagocytosis assay, we confirmed that almost all MGLs internalized latex beads after 5 h of incubation and similarly fluorescently labeled amyloid-β aggregates (*SI Appendix*, Fig. S6*A*). Phagocytic cells express high levels of NOX2, a NADPH oxidase (NOX) subtype (2, 28). The sole function of NOX2 in microglia is to generate ROS, a short-lived intermediate that is important in redox signaling circuits, shaping the different activation phenotypes of microglia (2). To confirm this core feature of microglial function, which is similar to that in MACs, we performed ROS live-cell imaging at baseline and upon stimulation with the NOX activator phorbol 12-myristate 13-acetate (PMA), showing similar ROS production rates in MGLs and MACs. Treatment with the NOX2-specific inhibitor gp91-TAT resulted in almost complete blockage of the PMA-induced increase in ROS production rates in both cell types, confirming their common ability to generate high rates of NOX2-derived ROS (Fig. 4*A*).

Microglia and other myeloid cell types showed altered and overall increased cytokine secretion upon stimulation with lipopolysaccharide (LPS) and interferon (IFN)-γ. MGLs show differing cytokine secretion profiles compared with both unstimulated controls (*SI Appendix*, Fig. S6*B**)* and MACs and monocytes in microglia medium (MonoNGD) (Fig. 4*B*). While significantly higher concentrations of tumor necrosis factor (TNF), IL-6, and IFN-γ were detected in stimulated MGL cultures, stimulated MACs and monocytes secreted larger amounts of IL-1b, IL-1RA, and IL-10, which were barely or not detectable in MGLs (Fig. 4*B* and *SI Appendix*, Fig. S6*B*). This finding is in line with the characterization of MGLs from previous differentiation protocols also reporting a lack of secretion of these cytokines upon LPS stimulation (9, 27).

After identifying the microglia-like identity of our MGLs and benchmarking them against human microglia ex vivo and peripheral MACs, we further investigated microglial functions. LPS stimulation in rodent microglia leads to an immuno-metabolic switch involving reduced oxidative phosphorylation and increased glycolysis (3). We measured the oxygen consumption rate in unstimulated versus LPS-stimulated MGLs using a seahorse analyzer. The significantly reduced oxygen consumption rate after stimulation indicated decreased oxidative phosphorylation and metabolic switching toward increased glycolysis (*SI Appendix*, Fig. S6*C*). The proliferation rate of MGLs is strongly decreased compared with MGL precursors, with only 1.7% of MGLs expressing KI-67 (*SI Appendix*, Fig. S6*D*).

Mass spectrometry of MGL lysates identified proteins involved in key microglia cell processes, including immune response, oxidative stress response, and IFN-γ, IL-1, and TGF-β signaling, and proteins associated with disease-related microglia responses, including Alzheimer’s disease, Parkinson’s disease, and amyotrophic lateral sclerosis, thus confirming MGL functionality (*SI Appendix*, Fig. S7 *A* and *B*). The 1.927 most abundantly expressed proteins in MGLs were included in a single enrichment analysis, performed by comparing the Gene Ontology (GO) terms of the MGL proteome with those of a randomly generated, equal-sized human proteome. The clustering revealed a dominant set of microglia-relevant terms, further supporting their similarity to primary microglia. Further prominent GO terms appearing within this analysis are connected to inflammation-related processes in activated microglia phenotypes and comprised terms like “cellular response to oxidative stress”, “TNF-mediated signaling pathway”, and “IL-1β-mediated signaling pathway” (*SI Appendix*, Figs. S8 and S9).

MGLs perform classical phagocyte functions comparable to MACs. To distinguish them from each other on a functional level, we performed calcium live-cell imaging using adenosine diphosphate (ADP) and adenosine triphosphate (ATP). Only microglia, but not MACs, express the purinergic receptor P2Y12 responsible for an ADP-induced Ca^2+^ influx into the cytoplasm (27). MGLs react with a Ca^2+^ influx upon stimulation with either ADP or ATP, whereas MACs lacking P2Y12 expression only react with a Ca^2+^ transient upon ATP, but not ADP, exposure (Fig. 4*C*).

### MGLs in Coculture with Tau-Mutant Neurons Adopt a Dystrophic Phenotype.

Next, we established a human in vitro model for studying the role of microglia in tauopathies. To this aim, we used a previously reported hiPSC tauopathy model, consisting of two different hiPSCs lines with distinct mutations in the *microtubule-associated protein tau* (*MAPT*) gene, causing hereditary forms of frontotemporal lobar degeneration (FTLD)-tau (*MAPT*^N279K^ [exon 10] and *MAPT*^V337M^ [exon 12]), one wild-type, age-matched control (CTRL1), and one isogenic control of the N279K-mutant hiPSC line (CTRL2) (28, 29). The N279K mutation causes aberrant splicing of exon 10, resulting in higher levels of 4R tau isoforms, which are more prone to tau aggregation (30). In contrast, the V337M mutation results in decreased interaction of the tau protein with microtubules (31). Both mutations result in hyperphosphorylation of the tau protein, leading to destabilization of microtubule assembly (32, 33).

**Fig. 5.**
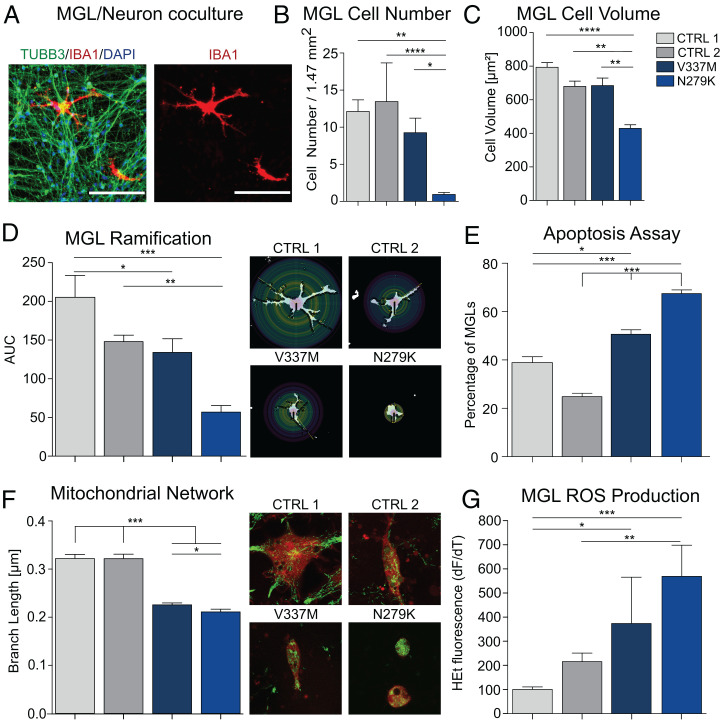
MGLs elucidate the role of microglia in a tauopathy model. (*A*) Coculture of cortical neurons and MGLs. (*B*) MGL cell counts in coculture with two neuron control lines (light gray: WT; dark gray: isogenic control of MAPT^N279K^) and neurons carrying the MAPT^V337M^ (dark blue) or MAPT^N279K^ (light blue) mutation, respectively. (*C*) MGL cell volume in coculture with neurons determined by area measurements. (*D*) Ramification of MGLs in coculture with neurons presented as mean area under the curve (AUC) values from Sholl analysis. (*E*) Apoptosis assay measuring caspase activity in microglia in coculture with neurons. (*F*) Average mitochondrial branch length determined by skeleton analysis in MGLs in coculture with neurons. (*G*) ROS production of MGLs in coculture with neurons. Statistical significance was determined using Bonferroni-corrected one-way ANOVA (*P < 0.05; **P < 0.01; ***P < 0.001; ****P ≤ 0.0001).

We generated pure cultures of cortical neurons from all four cell lines using a modified version of our previously reported neuron forward programming protocol (16). MGLs were added to the differentiated neuronal cultures (Fig. 5*A*), cocultures were cultivated in NGD medium, and after 1 wk, we investigated the survival rate of MGLs by counting IBA1^+^ cells and found a significantly decreased No. of MGLs in coculture with *MAPT*^N279K^ neurons (Fig. 5*B*). We repeated the experiment with an MGL-GFP reporter cell line (*SI Appendix*, Fig. S10), and flow cytometry analysis of GFP^+^ cells after 1 wk of coculture confirmed the decreased survival rate of MGLs in coculture with *MAPT*^N279K^ neurons (*SI Appendix*, Fig. S11). Morphological analysis of surviving MGLs in coculture revealed an altered microglia phenotype in coculture with mutant neurons. In specific, cell volume and the degree of ramification were significantly lower in MGLs cocultured with *MAPT*^N279K^ neurons, suggesting a dystrophic or activated disease-associated microglia phenotype (Fig. 5 *C* and *D*). A caspase assay demonstrated higher proportions of apoptotic MGLs in the coculture with *MAPT*^N279K^ neurons than in coculture with both control lines (Fig. 5*E*). The No. of caspase-positive cells was also slightly increased in MGLs cocultured with *MAPT*^V337M^ neurons. Another key feature of microglia activation in disease is increased mitochondrial fission as a metabolic activation marker (34, 35). To analyze mitochondrial networks and the degree of fusion and fission, we used a MGL reporter cell line and mitotracker staining to measure the average branch length in the mitochondrial network based on skeleton analysis (*SI Appendix*, Fig. S12). We found a significantly reduced mitochondrial branch length in MGLs cocultured with *MAPT*^V337M^ and *MAPT*^N279K^ neurons and a significantly shorter average branch length in the *MAPT*^N279K^ neuron coculture compared with the *MAPT*^V337M^ coculture (Fig. 5*F*). These changes were paralleled by increased ROS production by microglia in coculture with mutant neurons compared with controls (Fig. 5*G*). Taken together, these data demonstrate a secondary, genotype-dependent, disease-associated phenotype of wild-type microglia cocultured with tau mutant neurons.

### MGLs in 3D Brain Organoids Exhibit More In Vivo–Like Phenotypes.

**Fig. 6.**
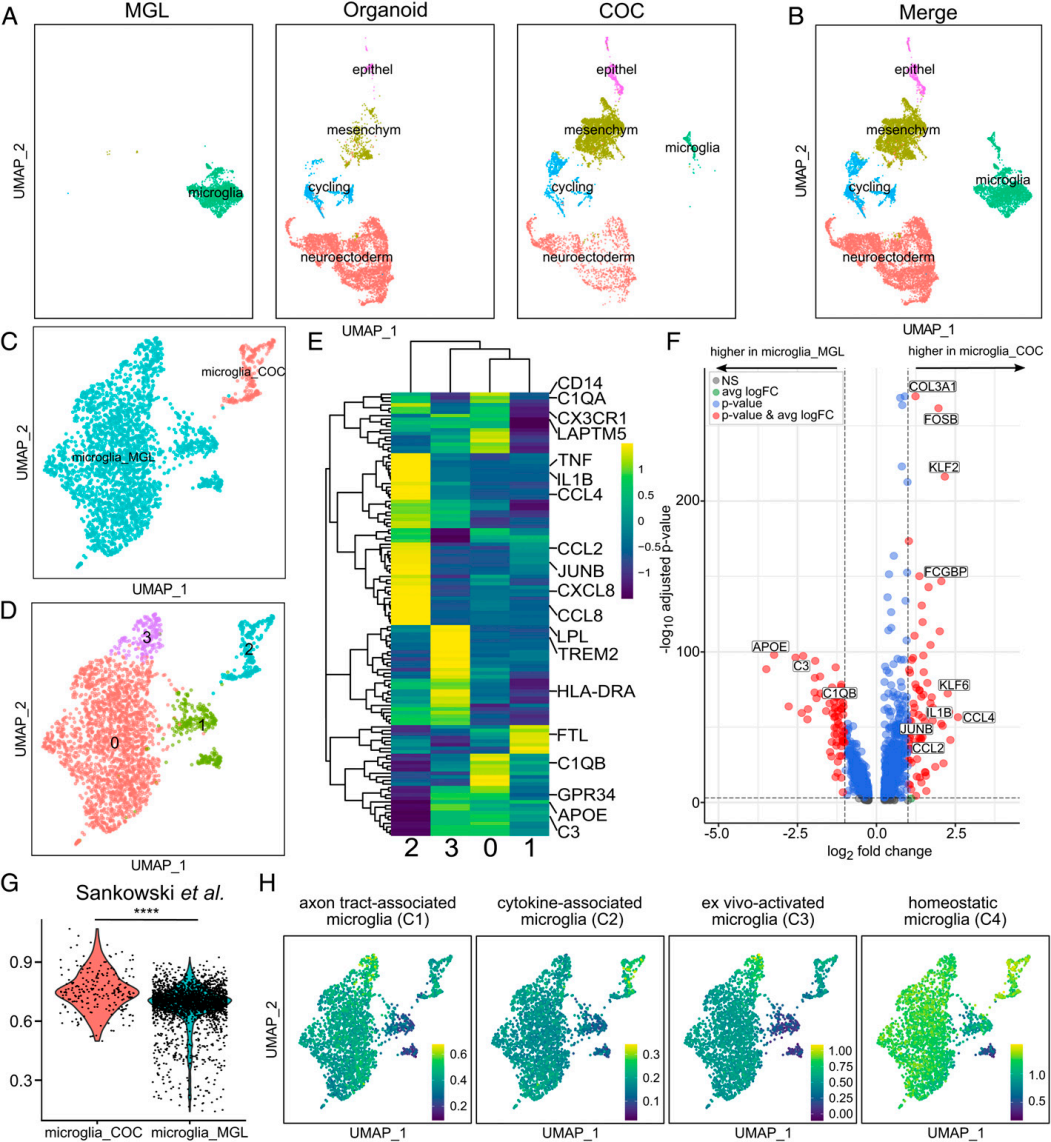
The single-cell MGL transcriptome changes in a complex cellular environment. (*A* and *B*) UMAP plot of 14,951 single-cell transcriptomes from three conditions: 2D MGL monoculture (MGL), organoids without microglia (organoid), and organoids cocultured with MGLs (COC) split by condition (*A*) or merged (*B*). (*C*) UMAP plot of 2,652 single-cell transcriptomes. MGLs in monoculture (microglia_MGL) compared with MGLs that were cocultured with organoids (microglia_COC). (*D*) UMAP plot of the same 2,652 single-cell transcriptomes, including microglia_MGL subclustering (clusters 0, 1, and 3) and microglia_COC (cluster 2). (*E*) Heatmap showing expression of a set of differentially expressed (DE) genes of primary human microglia (Table S3 in Sankowski et al. (38)) in microglia_MGL subclusters and microglia_COC (as defined in *D*). The values were scaled column-wise. (*F*) Volcano plot showing DE genes in microglia_MGL versus microglia_COC (as defined in *C*). The thresholds were set to a log_2_ fold change of 1 and a negative log_10_ *P* value of 3. Selected genes are labeled. The complete list of DE genes can be found in Dataset S2. (*G*) Violin plot displaying the global gene-set scores of microglia_COC (red) versus microglia_MGL (blue). The module scores were calculated from DE gene sets of primary human microglia (Table S3 in Sankowski et al. (38); the complete gene lists can be found in Dataset S3). *P* values were computed using the Wilcoxon rank sum test (****P ≤ 0.0001). (*H*) Feature plots depicting gene-set scores of the different microglia clusters based on previously defined clusters of microglia (51).

Finally, we developed a 3D coculture of MGLs in organoids. For this, we added MGLs to the culture medium of preformed cerebral organoids that had been differentiated for 30 d according to the original Lancaster protocol (36). Following another 30 d of coculture, organoids were harvested. Immunohistochemistry demonstrated the presence of ramified IBA1^+^ cells in both superficial and deeper layers of the organoid, demonstrating survival and migration of MGLs within the preformed organoid (*SI Appendix*, Fig. S13). We next performed single-cell RNA sequencing (scRNA-seq) by pooling and dissociating organoids to single cells and FACS for viable cells for 3′ microfluidics-based scRNA-seq (Chromium 10x). Three groups were compared: (i) MGLs in vitro (2,436 cells, 4,204 median genes per cell), (ii) cerebral organoids (6,642 cells, 3,720 median genes per cell), and (iii) cerebral organoids with MGLs (5,873 cells, 4,204 median genes per cell). Uniform manifold approximation and projection (UMAP) analysis demonstrated a relatively homogenous MGL population in the 2D monoculture (Fig. 6*A*). Organoids consisted of several major clusters, including a large neuroectodermal cell population (*NNAT*, *NCAM1*, and *GBX2*), alongside epithelial (*KRT19* and *CLDN4*), mesenchymal (*THY1*, *FN1*, and *VCAM1*), and cycling cells (*CDC45*, *CDK1*, and *CDC20*) (Fig. 6*A* and *SI Appendix*, Fig. S14 and Dataset S1). No cells transcriptionally resembling microglia were observed. In contrast, organoids with MGLs contained the same main clusters plus cells expressing core microglia genes (*HEXB*, *CSF1R*, and *CX3CR1*, Fig. 6 *A* and *B* and *SI Appendix*, Fig. S14). Interestingly, cells in the microglia cluster in organoids segregated from their counterparts in the 2D monoculture (Fig. 6*C*). Subclustering of all MGLs (including in vitro MGLs and MGLs in organoids) revealed four different clusters showing three subclusters of MGLs (c0, c1, and c3) and the MGL in organoid cluster (c2) (Fig. 6*D* and Dataset S5). Clusters 0 and 2 showed high expression of the classical myeloid and microglia signature genes (e.g., *CD14*, *C1QA*, and *CX3CR1*), while cluster 2 also showed high expression of proinflammatory cytokine genes (e.g., *IL1B*, *TNF*, and *CXCL8*) and chemokine genes (e.g., *CCL2*, *CCL4*, and *CCL8*) (Fig. 6*E*, also see Fig. 6*F*). In contrast, the smaller cluster 1 exhibited features of dystrophic microglia indicated by high expression of iron storage proteins (*FTL*) (37), while cluster 3 showed high expression of inflammatory and metabolism genes (*TREM2*, *HLA-DRA*, *APOE*, and *LPL*) (Fig. 6 *D* and *E*).

Genes that were significantly upregulated in MGLs in organoids compared with MGLs (*n* = 813) included many cytokines and chemokines (e.g., *IL1B*, *CCL2*, and *CCL4*), while downregulated genes encompassed other inflammation-related genes that are involved in different microglia signaling pathways, which were also identified in our MGL proteome analysis (*n* = 694, e.g., *APOE*, *C3*, and *C1QB*) (Fig. 6*F* and *SI Appendix*, Fig. S7 *A* and *B* and Dataset S2). Altogether, this suggests that distinct types of activated microglia predominate in different culture conditions.

Recently, Sankowski and colleagues defined eight major microglia clusters in the human brain (38). MGLs in organoids express genes from all eight clusters, whereas the in vitro counterparts preferentially upregulated genes from Sankowski clusters 2, 3, 6, and 7 (*SI Appendix*, Fig. S15). In total, we detected a significantly higher expression of previously published primary microglia gene signatures in our MGLs conditioned in organoids (microglia_COC) than in vitro (microglia_MGL) (Fig. 6*G*), indicating a broader range of microglia subtypes approximating the situation in the human brain, when cultured within organoids compared with the in vitro culture (*SI Appendix*, Fig. S15 and Dataset S3).

Additionally, we performed a comparable analysis with the four relevant microglia clusters of a study conducted by Popova et al. that aimed to find the most suitable conditions for microglia studies in vitro. Popova and colleagues concluded that microglia in organoids most closely resemble in vivo microglia (51). Interestingly, we found a significantly higher similarity of MGLs in coculture with organoids with “cytokine-associated microglia” and “homeostatic microglia” as shown in feature plots of the gene scores of the individual clusters (Fig. 6*H*) and related violin plots, including statistics of the module score (*SI Appendix*, Fig. S16). On the other hand, the gene-expression profile of our MGLs in vitro is more similar to ex vivo–activated microglia. Axon tract–associated microglia show similar expression levels in our in vitro MGLs and in coculture (Fig. 6*H* and *SI Appendix*, Fig. S16 and Dataset S4).

We next investigated which cell–cell interactions controlled the microglia phenotype in organoids. To this aim, we performed a cell-communication analysis between the MGLs and neuroectoderm clusters using CellPhoneDB (39). We identified genes that were significantly enriched in cellular interactions and differentially expressed in MGLs in organoids versus in vitro (e.g., *CCL3*, *GAS6*, *TNF*, and *VEGFA*) (Dataset S6). Interestingly, GO-term analysis demonstrated a role for many of the upregulated microglia surface proteins in brain development, such as “neurogenesis”, including “positive regulation of oligodendrocyte differentiation” and “dendrite arborization”, “positive regulation of axon regeneration”, and “axon extension” (Dataset S7). These findings support the importance of microglia in shaping the developing CNS. The top hit “VEGF-activated Neuropilin pathway” relates to the expression of neuropilin 1 (NRP1) in microglia, which has been studied in various contexts: NRP1-expressing microglia play a role in CNS vascularization (40), and they promote oligodendrocyte precursor proliferation during development and remyelination (41).

Taken together, single-cell analysis of microglia in organoids demonstrated a transcriptional shift toward in vivo–like microglial phenotypes containing subtypes of all predefined clusters opposed to in vitro MGLs only representing a part of the variety of microglia subtypes. These results must be considered in applications of microglia-containing brain organoids for developmental and disease-modeling studies.

## Discussion

In summary, we present a deterministic forward programming protocol for the generation of hiPSC-derived microglia with unprecedented efficiency. This protocol enables robust, scalable production of pure bulk quantities of MGLs. Compared with other previously published protocols (reviewed in ref. (10)), enrichment steps, i.e., FACS, or mechanical trituration of intermediate progenitor cell populations are not required. Moreover, defined, i.e., serum-free media compositions are applied, and the protocol duration is significantly shortened to 16 d. We confirmed that MGLs resemble microglia transcriptionally, functionally, and metabolically, including phagocytosis, cytokine secretion, ROS production, and ADP-evoked calcium transients. Bulk RNA sequencing analysis demonstrated high similarities between our programmed MGLs, previously reported MGLs derived from hPSCs by classical differentiation, and primary human microglia. In contrast, MGLs clustered away from other myeloid phenotypes, including monocytes, DCs, and MACs. Together, these data strongly support the microglial identity of our MGLs. Future studies should delineate differences in the RNA-expression profiles of blood-isolated monocytes versus MGLs in long-term coculture with neurons or brain organoids to confirm the distinct developmental origins.

From a technical perspective, the optimized inducible overexpression system enables overexpression of both master TFs in all cells of the initial hiPSC cultures; this modality of transgene delivery is the prerequisite for deterministic programming without the need of subsequent progenitor enrichment steps. The choice of the TFs PU.1 and C/EBPβ relates to their well-established role during the entire microglia lineage, as demonstrated by development knockout and large-scale, human-profiling studies. In contrast, other reprogramming factor candidates are associated with disadvantages, including less evidence for its capacity as pioneering factor (such as IRF8) and less specificity for the myeloid lineage compared with other leukocyte subsets (such as RUNX1) or the microglia lineage compared with other myeloid cells (such as C/EBPα) (42). Finally, it is well established from previous forward programming studies that the cellular target phenotype is the result of a timed interplay of TFs and extracellular cues. Therefore, we optimized the media composition for posterior primitive streak patterning, primitive hematopoiesis, yolk-sac MAC, and microglia maturation, respectively. Our media complexity-reduction trials indicated that cues for posterior primitive streak/extraembryonic mesoderm patterning were essential for the programming success. A most-recent study demonstrated development of MGLs within human cortical organoids upon overexpression of the TF PU.1 (43). Possibly, the cellular environment within the organoid mimics developmental stages of the CNS formation and microglia emergence and obviates the need for C/EBPβ as a second reprogramming factor.

The current method is explicitly designed for programming unlimited amounts of pure bulk quantities of MGLs. Another technical frontier is the direct conversion of easily accessible patient-derived cells (e.g., fibroblasts or blood cells) to MGLs, as this would enable the study of age-related changes without erasure of epigenetic marks that typically results from reprogramming to pluripotency. Importantly though, inherent to such lateral programming trajectories, the target cell populations will be smaller and heterogeneous due to the low proliferative capacity of both starting and target cells and the need for incomplete transfection methods for transgene overexpression.

To investigate the role of MGLs in neurodegenerative diseases, we developed a human FTLD in vitro model in which hiPSC-derived *MAPT*-mutant neurons were cocultured with MGLs. Previous studies have shown that microglia are spatially correlated with tau pathology in the tauopathies (44, 45). In autopsy studies of patients with FTLD, microglia activation has been shown most prominent in *MAPT*-mutation carriers compared with other genetic (*GRN* and *C9ORF72*) or sporadic forms of FTLD (46). Recent in vitro studies using mouse neuronal cultures demonstrated that exogenously applied tau induced mitochondrial and homeostatic calcium and ROS changes in neurons, leading to increased cell death. Similar findings were observed in hiPSC-derived *MAPT*-mutant neurons causing FTLD (47, 48). Both studies focused on neuronal phenotypes, which is inherent to the problem obtaining human microglia. It thus remained unclear whether endogenous mutations leading to FTLD can lead to secondary microglial phenotypes, which is to be expected, given the prominent role of microglia in FTLD. We found reduced MGL cell Nos., a higher amount of apoptotic MGLs, and increased mitochondrial network fragmentation but higher rates of ROS production in MGLs cocultured with *MAPT*^N279K^ neurons and similar but less pronounced changes in cocultures with *MAPT*^V337M^ neurons, compared with control cell lines. These results point to a prominent microglial activation in our in vitro FTLD models. Our findings show that neuronal mutations in an in vitro FTLD model induce a prominent secondary microglial phenotype, thus offering a plethora of opportunities to study microglia–neuron interactions in human coculture models. Follow-up studies with MGL–brain organoid cocultures will provide deeper insights into cellular interactions and disease mechanisms. Our study demonstrates that cultivation of MGLs in cerebral organoids is possible for significant periods of time, paving the way for significant microglial maturation effects and further tauopathy studies.

Several studies have shown that hiPSC-derived MGLs adopt an in vivo phenotype following their transplantation into the brain of humanized, microglia-depleted mice (6, 49). For some biotechnological applications, a more-complex, fully human model system using brain organoids is desirable. Several recent studies have provided proof of principle that MGLs may be placed in coculture with organoids (19) and confirmed the importance of the interplay between different cell types in these culture systems (43, 50–52). Indeed, MGLs placed in coculture with cerebral organoids have most recently been reported to resemble in vivo microglia most closely and therefore represent a valuable tool for investigations of brain development or neurodevelopmental as well as neurodegenerative diseases (51). We investigated the effect of the organoid environment on MGLs by using scRNA-seq analysis, demonstrating that microglia in organoids approximate published primary human microglia datasets more closely and are associated with the broader range of microglia found in the human brain rather than the more-artificial in vitro phenotypes. Cell communication analyses underscored the important role microglia play in shaping neurogenesis during embryonic development. Future projects should investigate organoid microglia cocultures at later stages of organoid development or using more-defined organoid culture protocols and focusing on specific regions within the organoid. This will enable studying the influence of greater levels of organoid complexity, including its effect on the inflammatory signature of microglia in organoids.

In conclusion, the presented versatile microglia platform enables the most rapid and efficient generation of pure bulk quantities of human microglia from hiPSCs to date, tailored by the interplay of transient overexpression of PU.1 and C/EBPβ and extracellular cues. Robust 2D and 3D in vitro coculture systems allow in vitro disease modeling and delineation of cell–cell interactions in complex culture systems, opening the door for future studies on long-term coculture experiments and complex neurodegenerative disease models.

## Materials and Methods

A detailed description of experimental procedures and analyses is provided in *SI Appendix, Detailed Experimental Procedures*. Briefly, hiPSCs were engineered using our previously reported dual GSH-based inducible transgene overexpression system to enable transient expression of the TFs PU.1 and C/EBPβ. Transgene expression was induced by addition of Dox to the culture media. Pure populations of MGLs were obtained after 16 d. MGLs were extensively characterized in 2D monocultures, 2D neuron cocultures, and 3D brain organoids.

## Supplementary Material

Supplementary File

Supplementary File

Supplementary File

Supplementary File

Supplementary File

Supplementary File

Supplementary File

Supplementary File

## Data Availability

The single-cell RNA-sequencing data reported in this paper have been deposited in the Gene Expression Omnibus (GEO) database, https://www.ncbi.nlm.nih.gov/geo (accession no. GSE207608) (53). Bulk RNA-seq data are available under the BioProject Accession No. PRJNA881328 (https://www.ncbi.nlm.nih.gov/bioproject/881328) (54). Proteome data are available via ProteomeExchange with identifier PXD024649 (55). [single-cell RNA-sequencing data [Gene Expression Omnibus (GEO)] bulk RNA-seq data [BioProject] raw data [Proteome Exchange]] data have been deposited in [Gene Expression Omnibus (GEO) Bio Project ProteomeExchange] (GSE207608 PRJNA881328 PXD024649).
