# Supplementary material for: Deterministic programming of human pluripotent stem cells into microglia facilitates studying their role in health and disease
Source: Proc Natl Acad Sci U S A. 2022 Oct 17;119(43):e2123476119. doi: 10.1073/pnas.2123476119 (PMC9618131; doi:10.1073/pnas.2123476119)
Supplement: Supplementary File [file pnas.2123476119.sapp.pdf]

PNAS

Supplementary Information for
Deterministic programming of human pluripotent stem cells into microglia facilitates studying their role in health and disease.

Anna M. Speicher, PhD[a], Lisanne Korn[a]; Júlia Csatári[a]; Laura Gonzalez-Cano, PhD[b]; Michael Heming, MD[a]; Christian Thomas, MD[c]; Christina B. Schroeter, MD[a]; David Schafflick[a]; Xiaolin Li, PhD[a]; Lukas Gola[a]; Alexander Engler[d]; Thilo Kaehne, PhD[d]; Ludovic Vallier, PhD[e,f]; Sven G. Meuth, MD PhD[a]; Gerd Meyer zu Hörste, MD[a]; Stjepana Kovac, MD PhD[a]; Heinz Wiendl, MD[a]; Hans R. Schöler, PhD[b,g]; Matthias Pawlowski, MD PhD[a,1]

Corresponding author: Matthias Pawlowski, MD PhD
Email: matthias.pawlowski@ukmuenster.de

This PDF file includes:

Supplementary text
A. Detailed Experimental Procedures
B. Materials
Figures S1 to S16
Legends for Datasets S1 to S7
SI References

Supplementary Information Text

A. Detailed Experimental Procedures

1. Human iPSC lines and maintenance of pluripotency cultures

We used two independent, previously established, well-characterised, wild-type hiPSC lines: F13B hiPSCs were generated as part of the HipSci-project from adult skin fibroblasts by using Sendai virus reprogramming vectors at the Wellcome Trust Sanger Institute, United Kingdom (hPSCreg: WTSIi269-A) (1). C2-1 hiPSCs were generated from neonatal skin fibroblasts by using retrovirus vectors at the Max-Planck-Institute for Molecular Biomedicine, Germany (hPSCreg: MPIi001-A) (2). The study was approved by the local ethics committee (AZ 2019-390-f-S). All hiPSC lines were cultivated in a commercially available pluripotency maintaining medium (StemMACS iPS Brew XF, human; Miltenyi) in 6-well plates coated with growth-factor reduced Matrigel (Corning). The cells were kept in a humidified incubator at 37°C and 5% CO_2. Fresh medium was added daily, and passaging was performed every 4-5 days using Accutase (Sigma).

2. Human ex vivo microglia

Human ex vivo microglia were obtained from the Netherlands Brain Bank (NBB). They stem from a non-demented female aged 60 years. Microglia were isolated post mortem, following the protocol by Mizee et al. (3). Briefly, autopsy was performed with approximately 6h post-mortem delay. Subcortical white matter and occipital cortex grey matter were mechanically dissected and enzymatically dissociated using collagenase I or trypsin for 60 min. Enzyme activity was quenched using fetal calf serum (FCS) and cell suspension was centrifuged. The cell pellet was resuspended in Dulbecco's Modified Eagle's Medium (DMEM) containing FCS and antibiotics and passed through a 100µm sieve. With Percoll gradient centrifugation microglia were finally isolated. After two washing steps with DMEM containing FCS and antibiotics, positive selection of microglia with anti-CD15 and subsequently with anti-CD11b magnetic microbeads was performed using magnetic activated cell sorting (Miltenyi Biotec). Isolated primary microglia were resuspended in TRIsure buffer and stored at -80°C. Subsequent RNA isolation was performed using phase separation by addition of chloroform and centrifugation and following RNA precipitation in isopropanol.

3. Molecular Cloning

The hROSA26 donor plasmid and the Cas9n and hROSA26 guide RNAs expressing plasmids were used as described previously (4, 5). AAVS1 ZFN expression vectors were used from the same source (4, 5). The AAVS1 donor vector was constructed by replacing EGFP with a bicistronic PU.1-T2A-C/EBPβ expression cassette. PU.1 was amplified by PCR from pWPT-hSPI1 which was a generous gift from Thomas Moreau (NHSBT Cambridge Centre). C/EBPβ was amplified by PCR from a C/EBPβ expression plasmid which was purchased from Dharmacon. During PCR amplification we inserted T2A and EcoRI/SpeI restriction sites. The vector backbone was restricted using EcoRI and SpeI to remove EGFP and all three fragments were purified by extraction from an agarose gel and were combined by Gibson Assembly (New England Biolabs).
For the generation of the CLYBL-mCherry donor vector pC13N-iCAG.copGFP (Addgene: #66578; (6)) we first replaced the kanamycin resistance cassette by a hygromycin resistance cassette. Therefore, the vector was restricted with XmaJI and CsiI and hygromycin was PCR amplified from pSH231-EF1-RFP-HYGRO (Addgene: #115145; (6)). Both were purified by agarose gel extraction and ligated using Gibson Assembly. In a second step, copGFP was replaced by mCherry by cutting pC12N-iCAG.copGFP_hygro with Bsp1407 and MluI. MCherry was PCR-amplified from CLYBL_hOPM (Addgene: #112499). Both fragments were again purified by agarose gel extraction and ligated by Gibson assembly.

4. Nucleofection

Targeting of the human orthologue of the mouse ROSA26 locus (hROSA26) in hiPSCs was performed as we described recently (5). Briefly, a single cell suspension was generated by incubation of cells with Accutase for 5 min at 37°C. 2 x 10^6 cells were resuspended in 100µl nucleofection solution containing 4 µg of each plasmid (two plasmids encoding the hROSA26 guide

RNAs and CAS9 nickase and the donor plasmid containing the CAG-rtTA transgene) and electroporated by using the program B-16 of the Amaxa nucleofector. After 5 min incubation time at room temperature (RT), 500µl of warm iPS medium containing Rho-associated protein kinase (ROCK) inhibitor Y-27632 (tebubio) was added into the cuvette. After another 5 min incubation time at RT nucleofected cells were plated on Matrigel-coated culture dishes (10 cm) in medium containing ROCK-inhibitor. 24h after nucleofection ROCK-inhibitor was removed and cells were cultured in iPS medium. After approximately 5 days, when non-confluent colonies had emerged, neomycin-resistant cells were selected by adding G418 (100µg/ml) for 5-7 days. G418-resistant colonies were individually picked, expanded and analyzed by genotyping.

Established hROSA26_CAG-rtTA cell lines were targeted using the new AAVS1 donor plasmid with the inducible transgene cassette containing the two TFs (see above). Targeting was again performed by nucleofection, and antibiotic selection was performed using puromycin (0,3µg/ml). After colony picking, the clones were analyzed by genotyping and immunocytochemistry against the two inducible transgenes (PU.1 and C/EBPβ) after 24h doxycycline induction.

For the generation of microglia reporter cell lines, established dual hROSA26/AAVS1 inducible cell lines were subjected to a third GSH targeting in the CLYBL locus. For this, we used a donor plasmid containing either copGFP or mCherry and a hygromycin resistance cassette. For site specific integration we used two TALEN plasmids (addgene #62197 & #62197; (6)). The three plasmids were nucleofected as described for the two previous GSH targeting steps. After transfection, antibiotic selection was carried out using hygromycin (50µg/ml). Picked clones were checked for homogenous reporter protein expression by fluorescence microscopy and flow cytometry.

5. Microglia forward programming protocol of GSH-targeted hiPSCs

Targeted human iPSCs were cultured to approximately 75% confluency and then detached using Accutase for 5 min at 37 °C. The cells were collected using a serological pipette and centrifuged at 300 x g for 5 min. The cells were resuspended in stem cell medium (iPS Brew XF, Miltenyi) containing ROCK inhibitor (10 µM) and seeded at a density of 2×10^5 cells per 6-well on a Matrigel-coated (80 µg/ml; o/n at +4°C; no wash) culture dish. After 24h the medium was switched to stem cell medium without ROCK-inhibitor. 48h after initial seeding the cells were cultivated in DMEM/F12 basal medium (DMEM/12, B27, N2, Glutamax, P/S) supplemented with the listed cytokines: Day 0 of induction: Activin A (10 ng/ml), BMP4 (10 ng/ml), FGF (20 ng/ml), VEGF-A (50 ng/ml), LY (10 µM), CHIR (8 µM) and Dox (1 µg/ml). Day 2 and Day 4 of induction: SCF (50 ng/ml), IL-3 (20 ng/ml), IL-6 (10 ng/ml), M-CSF (50 ng/ml), FGF (10 ng/ml), doxycycline (1 µg/ml). Day 6 and Day 8 of induction: CSF1 (50 ng/ml), Dox (1 µg/ml). By day 10 of induction, cells had detached from the culture dish and were free floating in the supernatant of the culture. These floating cells were collected on day 10 and replated on poly-L-lysine (PLL)-coated (5 µg/ml; 30min at RT; 2x wash with Phosphate-Buffered Saline (PBS)) 12-wells in a density of 2×10^5 cells per 12-well. From day 10 onwards, cells were cultivated in a basal medium previously defined by Muffat et al. (7) supplemented with IL-34 (100 ng/ml), CSF1 (10 ng/ml) and TGF-β (50 ng/ml), whereas Dox was omitted from the culture media. Media changes were performed every 3-4 days until analysis. After 16-20 days of total culture duration (i. e. 6-10 days after replating), cells were used for analysis.

6. Immunocytochemistry

Cells on coverslips or in a culture plate were fixed in 4% paraformaldehyde (PFA) for 15 min at RT and washed three times using DPBS [-]$CaCl_2$ [-]$MgCl_2$ (PBS-/-). Subsequently, cells were blocked with a blocking solution of PBS-/- containing 10% serum (Sigma Aldrich) and 0.3% Triton X-100 (Sigma Aldrich) for 30 min at RT. The cells were then incubated with a primary antibody solution containing 2% serum, 0.1% Triton X-100 and the appropriately diluted antibody over night at 4°C. After washing the cells three times with PBS-/- the secondary antibody solution, containing 1% serum, 0.1% Triton X-100 and the appropriately diluted secondary antibody and 4',6-diamidino-2-phenylindole (DAPI, 1µg/ml, Thermo Fisher Scientific), for one hour at RT in the dark. After three washing steps using PBS-/- cells were analyzed using a fluorescence microscope (Leica, DMI6000 B).

7. Flow cytometry

For the flow cytometric analysis of surface marker expression, the cells were collected as single cell suspension and transferred through a cell strainer (40µm). Cells were washed in FACS buffer consisting of PBS-/-, 0.5% BSA and 2 mM EDTA and then incubated in FACS buffer containing the extracellular antibodies in an appropriate dilution for 15 min at RT in the dark. Cells were again washed in FACS buffer, resuspended in 100-200µl of FACS buffer and then analyzed on a Gallios Flow cytometer (Beckman Coulter).

8. Phagocytosis assay

a. Fluoresbrite microbead

To investigate the phagocytic activity of MGLs we added fluorescently labelled latex beads in a ratio of 100 beads/cell. Analysis was performed after 1, 3, 5, and 24h of incubation. For flow cytometric analysis, we detached the cells after the respective incubation time, washed 3x with PBS-/-, resuspended the cells in FACS buffer and performed analysis using a Beckman Coulter Gallios Flow Cytometer. Additionally, cells were deposited on microscope slides using a cytospin. Cells were mounted with Fluoromount containing DAPI and imaged with an inverted microscope (BZ-9000 BioRevo; Keyence).

b. TAMRA labelled Amyloid-β

$A\beta_{1-42}$ (AnaSpec) was reconstituted to a stock solution of 1mg/ml in 1% NH_4OH as stated in the data sheet, further diluted to 100µg/ml using endotoxin-free water. The solution was vortexed thoroughly and incubated at 37°C for 7days to form fibrils. Prior to cell exposure fAβ was thoroughly mixed. After 24h cells were collected, washed with PBS-/- and analyzed using flow cytometry. Another part of the cells was fixed in 4% PFA and counterstained with the microglia marker IBA1.

9. Cytokine secretion assay

For the investigation of cytokine secretion, MGLs, MACs and monocytes were stimulated with LPS (100 ng/ml) and IFN-γ (20 ng/ml) for 48h. The supernatants were collected, and the cytokine secretion assay was performed following manufacturer's instructions (LEGENDplex human macrophage/microglia panel, Biolegend).

10. ROS measurements and calcium imaging

For live cell imaging, an epifluorescence inverted microscope equipped with a 40x oil-immersion fluorite objective was used. Fluorescence data was analyzed with MetaFluor Fluorescence Ratio Imaging Software (Molecular Devices, LLC, Canada/US) and Origin, Version 2019 (OriginLab Corporation, Northampton, MA, USA). Dyes were diluted in artificial cerebrospinal fluid (120 mM NaCl, 2.5 mM KCl, 1.25 mM NaH2PO4, 22 mM NaHCO3, 25 mM glucose, 2 mM CaCl2, 2 mM MgSO4). MGLs were plated on PLL (5 µg/ml) coated coverslips (Ø 12 mm).

For detection of cytosolic ROS levels, cells were stained with 16 µM dihydroethidium (DHE, D11347, Invitrogen™) and measured immediately. Excitation light was provided by a LED lamp at 530 nm and emitted light was detected >670 nm. DHE fluorescence was measured with a frame interval of 5 seconds and fluorescence increase over time was calculated to determine the rate of ROS production.

To evaluate cytosolic calcium levels, cells were preincubated for 30 min with 5 µM Fura-2 (AM, F1221, Invitrogen™). During the experiment, cells were kept in artificial cerebrospinal fluid. The ratiometric calcium indicator was excited at 340 nm and 380 nm and emitted light was detected at 510/80 nm. The frame interval was set to 1 second.

11. Seahorse – Oxygen Consumption

One day prior to seeding, the Seahorse XFp cell culture plates were coated with poly-D-lysine (PDL) [50µg/ml] and incubated at 37°C without CO_2. Also, one day before the assay, sensor cartridges and surrounding chambers were hydrated with calibrant buffer (200 to 400 µl) and incubated overnight at 37°C without CO_2. On the following day, plates were washed three times with 500 µl PBS, cells were seeded in NGD medium at a density of 1 x 10^5 cells per well and incubated for 3 days at 37°C. Each plate contained three wells of MGLs that were stimulated with lipopolysaccharides (LPS) (100ng/ml) for 24 h and three wells MGLs without LPS as a control group. Cells were measured in artificial CSF containing pyruvate (1 mM). After signal stabilization

the cells were sequentially exposed to the mitochondrial stressors oligomycin (2 µM), FCCP (1 µM) and rotenone (0.5 µM), plus antimycin A (0.5 µM). OCR was determined using a Seahorse XFp Analyzer (Agilent), and assays have been analyzed with Wave Desktop software (Agilent).

12. Generation of cortical neurons and microglia-neuron coculture

Human cortical neurons were generated using a modified version of our recently published forward programming protocol based on NGN2 overexpression (5). Stem cell lines (8, 9) targeted with an inducible NGN2 expression cassette were induced with Dox (1 µg/ml) to turn on NGN2 transgene expression when cells had reached approximately 40-50% confluency. Cells were cultivated in neuronal medium (DMEM/F12, 2 % B27, 1 % N2, 1 % Pen/Strep, 10 ng/ml neurotrophin-3 (NT-3), 10 ng/ml brain-derived neurotrophic factor (BDNF), 0.2 mM cyclic adenosine monophosphate (cAMP), 200 µM ascorbic acid). Cortical neurons were placed into coculture with MGLs at day 14-28 at a 1:4 ratio.

13. Mitochondrial network analysis

To examine mitochondria in MGLs, cocultures of neurons and MGLs (plated on Matrigel approx. 4:1) were stained with 20 nM MitoTracker™ (M22426, Invitrogen™) for 30 min. Imaging was conducted with a Confocal Laser Scanning Microscope Leica SP8 equipped with a 63x oil-immersion objective. Reporter MGLs expressing mCherry were identified at 561 nm (emission >570 nm) and MitoTracker™ fluorescence was excited with a multiargon laser at 633 nm. Emitted light was gathered with a wavelength >670 nm and images were analyzed with ImageJ 1.52p (Wayne Rasband, National Institute of Health, USA) as described by Valente et al. (10). In short, fluorescent intensity threshold was manually set to allow optimal visualization of the mitochondrial network. MitoTracker fluorescence was transformed into a binary (black and white) output under visual control for adequate transformation. To further elucidate mitochondrial morphology, the binary was skeletonized, and single mitochondrial branch length was determined from this skeletonized mitochondrial network as described previously ((10); see Figure S16 for visual explanation of image processing). Average branch length of these single mitochondrial branches was determined as a surrogate marker for mitochondrial integrity and the degree of fusion and fission within the mitochondrial network.

14. Brain organoid generation & microglia-organoid coculture

Cerebral organoids were generated according to the original Lancaster protocol using the same wild-type hiPSC line used for the microglia derivation (11). 30 days old organoids were then co-cultured with mature MGLs. For ICC, coculture organoids were fixed overnight in 4% PFA, embedded in Optimal cutting temperature compound (OCT) and frozen prior sectioning using a standard cryostat. 20-µm sections were used for immunostaining after 3 or 30 days in co-culture. For scRNA sequencing, 100 organoids were pooled and dissociated using Accutase, followed by FACS for viable cells (using a co-staining of Calcein AM (ThermoFisher, C1430), Zombie NIR (Biolegend, 423105) and DAPI) and subsequent RNA isolation.

15. PBMC and monocyte isolation from human blood and differentiation into macrophages

Peripheral blood mononuclear cells (PBMCs) were isolated from donor blood by density gradient centrifugation with lymphocyte separation medium. Subsequently monocytes were isolated with magnetic bead isolation following manufacturer's instructions (Monocyte Isolation Kit II (indirect, labelling of non-monocytes); Miltenyi Biotech). Monocytes were immediately used for downstream applications, plated on PLL-coated culture dishes and cultivated in NGD medium or differentiated into macrophages by either cultivating them in RPMI medium containing 10% FCS, 1% Pen/Strep and M-CSF (MAC) or in XVIVO-10 medium containing GM-CSF (100 ng/ml) for serum-free macrophage differentiation (MACsf). After 5 days, cells were either used for further experiments and analysis or cultured in NGD medium for another 72 h prior to analysis.

16. RNA isolation and quantitative RT-PCR (qPCR)

RNA was isolated using the Qiagen RNeasy Mini Kit and the On-column DNase I Digestion Set (Sigma-Aldrich). Subsequent cDNA synthesis was performed using the Maxima First Strand cDNA

Synthesis Kit (Thermo Fisher Scientific). For qPCR the Maxima SYBR Green Master Mix was used (Thermo Fisher Scientific). qPCR reactions were run on a StepOne System (Applied Biosystems). All samples were analyzed in technical duplicates and normalized to the housekeeping gene glycerinaldehyd-3-phosphat-dehydrogenase (GAPDH). Results were analyzed with the ΔΔCt method.

17. Proteome analysis

Normalization: Cells were lysed in 200 µl lysis buffer consisting of 200mM HEPPS in 8M urea, pH 8.5 supplemented with 1x Halt Protease and Phosphotase Inhibitor (Thermo Fisher). Protein lysates were sonicated for 20 sec on 10 % power. After centrifugation of the samples at 1.400 rpm for 30 min at 4°C, the supernatants were transferred to new tubes. Subsequently, protein concentrations were determined using a commercial BCA kit (Thermo Fisher). Protein adjusted sample aliquots were subjected to sodium dodecyl sulfate–polyacrylamide gel electrophoresis (SDS-PAGE) to fine-adjust protein amounts for label free proteome analysis. After staining the gel with Coomassie Blue according to manufacturer's protocol the optical density of each sample lane was determined with a calibrated gel scanner in transmission mode and the relative protein amount was calculated.

Digestion and fractionation: Sample preparation for mass spectrometry was performed via in-solution digestion and strong cation exchange (SCX) fractionation. In brief, samples were four-fold diluted in 25 mM NH4HCO3, pH 8.0, and subsequently incubated with 5 mM dithiothreitol at RT for 1 h. Afterwards, reduced cysteine residues were carbamidomethylated via addition of 20 mM iodine acetamide at RT for 1 h. Proteins were digested by adding 2.5 µg trypsin (TrypsinGold, Promega, Madison, WI, USA) and incubated at RT overnight. Digestion was stopped by adding formic acid (FAc) to a final concentration of 0.5% and subsequently centrifuged at 15.000 x g at 4°C for 15 min. Resulting supernatant was subjected to a SCX column (SCX SpinTips, Protea Biosciences, Morgantown, USA) previously equilibrated with 60 µl acetonitrile (ACN) and washed with 0.1% trifluoric acid (TFA). After sample application SCX column was washed with 60 µl 0.1% TFA. Fractionation was achieved by stepwise elution with 60 µl of: 50 mM ammonium formiate, 20% ACN, 0.5% FAc; 75 mM ammonium formiate, 20% ACN, 0.5% FAc; 125 mM ammonium formiate, 20% ACN, 0.5% FAc; 200 mM ammonium formiate, 20% ACN, 0.5% FAc; 300 mM ammonium formiate, 20% ACN, 0.5% FAc and 5% ammonium hydroxide, 80% ACN. Eluted fractions were dried in a vacuum centrifuge.

Mass spectrometry: LC-MS/MS was performed on a hybrid dual pressure linear ion trap/orbitrap mass spectrometer (LTQ Orbitrap Velos Pro, Thermo Scientific, San Jose, CA, USA) equipped with an Ultimate 3000-nLC Ultra HPLC (Thermo Scientific, San Jose, CA, USA). Dried peptide fractions were dissolved in 10 µL 0.1% TFA and subjected to a 75 µm I.D., 25 cm PepMap C18-column, packed with 2 µm resin (Dionex, Germany). Separation was achieved by applying a gradient from 2% ACN to 35% ACN in 0.1% formic acid (FA) over a 130 min gradient at a flow rate of 300 nL/min. The LTQ Orbitrap Velos Pro MS exclusively used CID-fragmentation when acquiring MS/MS spectra, consisting of an orbitrap full MS scan followed by up to 20 LTQ MS/MS experiments (TOP20) on the most abundant ions detected in the full MS scan. The essential MS settings were as follows: full MS (FTMS; resolution 60,000; m/z range 400–2000); MS/MS (Linear Trap; minimum signal threshold 500; isolation width 2 Da; dynamic exclusion time setting 30 s; singly charged ions were excluded from selection). Normalized collision energy was set to 35%, and the activation time was set to 10 ms.

Data processing: Raw data processing and protein identification of the high resolution orbitrap datasets were performed with de novo sequencing algorithms of PEAKS Studio 8.0 (Bioinformatics Solutions Inc., Waterloo, Canada) using the SwissProt database. The false discovery rate was set to <1%.Raw data, complete identification listings and supplementary information are available via ProteomeExchange with identifier PXD024649.

GO-clustering and visualization:

The single enrichment analysis was performed by comparing the accumulated, annotated Gene Ontology terms (12) of the MGL proteome with those of a randomly generated, equal-sized human proteome. To calculate the significance of the difference between each proteome the fisher's exact was used and false positives were corrected using the Benjamini & Hochberg method (13). Utilizing

the underlying structure of the Gene Ontology, these significant different terms were clustered based on their child-parent relationship, grouping significant child terms with their significant parent terms.
Afterwards a graph was generated using the proteins as nodes that are connected by their significant parent terms by edges. This graph was then visualized with Gephi (http://gephi.org) using a force-directed graph drawing algorithm to calculate the layout, where virtual forces are applied to the nodes and edges. Whereas nodes generally push each other away, the edges pull them together – the more similar the proteins are to each other, the stronger the force of attraction. Therefore, defined by their shared GO terms, that are depicted as the connections (edges) in the graph, dense protein clusters are highly similar whereas dissimilar proteins show a spatial distance, because the number of shared terms (edges) are not sufficient to overcome the pushing forces of the proteins when the layout is calculated.
In Figure S7, scatter plots are shown depicting the expression of pre-selected protein groups throughout all three biological replicates. For that purpose, downstream bioinformatic analysis was performed using Perseus v 1.6.14.0 as previously described (14, 15). LFQ values were logarithmized (log2(x)) and missing values were replaced from normal distribution after deleting all proteins only detected in one out of three biological replicates. The data was searched against the integrated Perseus reference database for homo sapiens downloaded in March 2021 (http://annotations.perseus-framework.org).

18. Bulk RNA-sequencing

mRNA enrichment (NEB) was followed by a directional library preparation (NEB) and single-end sequencing on an Illumina NextSeq 500 instrument was performed at an average depth of 18.0 million reads. A total of 14772 expressed genes were detected per sample (range, 13017 - 16911). Transcript-level expression was quantified from the raw reads (FASTQ format) using the Gencode v35 annotation and the Salmon software package (version 1.3.0). Downstream analyses were performed in the R environment (version 4.1.0). Gene-level summarization of counts and combination of all sample data was carried out with tximport (version 1.20.0) and the tximport list was transformed into a DESeqDataSet with DESeq2 (version 1.30.1). A gene with 10 or more counts was considered expressed. Raw counts were transformed using variance stabilizing transformations (VST) function of DESeq2. A total of 14611 expressed genes were detected per sample (range, 13017 - 16911).

Integration with publicly available datasets

To compare different MGLs and effects of *in vitro* culture, we integrated publicly available datasets from three differentiation protocols (7, 16, 17), from Abud et al. (GSE89189, n=43), Douvaras et al. (GSE97744, n=24), and Muffat et al. (GSE85839, n=16). Moreover, we included RNA expression profiles from an extensive dataset of primary human microglia in vitro and ex vivo, and blood-isolated monocytes and macrophages (18) (Table S2 from (18), n=64). Processed RNA-seq data was normalized using the removeBatchEffect() function of the limma package (v3.5) specifying the datasets as batch. Normalized data were log transformed and a matrix with the 1,000 most variably expressed genes across all datasets was used as input to generate the MDS analysis using the plotMDS() function of the limma package.

19. Generation of single cell RNA libraries and sequencing

The samples were loaded onto the 10x Genomics Chromium Single Cell Controller with the Chromium Single Cell 3' Library and Gel Beads Kit v3. Library preparation was carried out according to the manufacturer's instruction using AMPure beads (Beckman Coulter). Sequencing was performed on an Illumina Nextseq 6000 with a 150-8-0-150 read setup. The bcl files were demultiplexed with cellranger mkfastq v3.1 and counted with cellranger count v3.1 according to the manufacturer's instructions.

20. Single cell RNA sequencing data analysis

Downstream analysis was performed with R v4.1 and Seurat v4 (19) based on the official vignettes. Briefly, low quality cells were removed by filtering cells with few genes (<200) or high genes (>7000) or high mitochondrial percentages (15%) for each sample individually. The data were normalized

using logarithmic transformation normalization, highly variable genes were identified, and the data were scaled regressing out mitochondrial percentage and sequencing depth. Principal component analysis was performed and the first 30 PCs were used for Uniform Manifold Approximation and Projection (UMAP). The clusters were determined in Seurat with the FindNeighbors and FindClusters function and annotated based on known marker genes. The steps were reperformed for the microglia subset. The top markers were identified with the FindMarkers function in Seurat based on the Wilcoxon rank sum test and an adjusted p value threshold (Bonferroni correction) of 0.05 and average log2 fold change of 0.25. Volcano plots were produced with Enhanced Volcano v1.10. The gene set scores were calculated with the AddModuleScore function in Seurat. Interactions between the neuroectoderm and microglia cluster were identified with CellPhoneDB v2.1.7 (20) following the official instructions. Statistical iterations were set at 1000 and the threshold of the cells expressing the ligand/receptor was set at 10%.

21. Statistical analysis

Statistical analysis was carried out with GraphPad Prism Version 5 and R v4.1.

B. Materials

Tissue Culture Consumables and Kits

Product	Company	Cat. No.
Accutase	ThermoFisher	A1110501
StemMACS iPS-Brew XF human	Miltenyi Biotech	130-104-368
Y-27632 Dihydrochloride (ROCK inhibitor)	Tebu-bio	10-2301-25mg
Matrigel, HC, GFR, LDEVfree	Corning	354263
DMEM/F12	ThermoFisher	11320074
N2 Supplement	ThermoFisher	17502048
B27 Supplement	ThermoFisher	17504044
Penicillin-Streptomycin	ThermoFisher	15140122
Recombinant Human/ Murine/ Rat Activin A	PeproTech	AF-120-14E
Recombinant Human BMP4 (E. coli derived)	PeproTech	120-05ET
Recombinant Human FGF-basic	PeproTech	100-18C
Recombinant Human VEGF-A	PeproTech	100-20A
LY-294002 hydrochloride	Sigma Aldrich	L9908-1MG
CHIR 99021	Tocris	4423
Doxycycline hyclate	Sigma Aldrich	D9891
Recombinant Human SCF	PeproTech	300-07
Recombinant Human IL-3	PeproTech	200-03
Recombinant Human IL-6	PeproTech	200-06
Recombinant Human CSF1 (M-CSF)	PeproTech	300-25
Recombinant Human IL-34	PeproTech	200-34
Poly-L-Lysine Hydrobromide	Sigma Aldrich	P1274-25MG
NaCl 5M	Sigma Aldrich	S5150-1L
L-Ascorbic Acid	Sigma Aldrich	A4403-100MG
AlbuMAX I Lipid-Rich BSA	ThermoFisher	11020021
Biotin	Sigma Aldrich	B4639-100MG
GlutaMAX Supplement	ThermoFisher	35050038
Sodium Pyruvate	ThermoFisher	11360039
Lactic Acid 85 % syrup	Sigma Aldrich	L1250-500ML
Neurobasal Medium	ThermoFisher	12348017
Neomycin (G-418 solution)	Sigma Aldrich	4727878001
Puromycin Dihydrochloride	ThermoFisher	A1113803
Human Stem Cell Nucleofector Kit 1	Lonza	VAPH-5012
Fluoresbrite Polychromatic Red Microspheres 1µm	Polysciences	18660-5
[Met]-beta-Amyloid(1-42),5-TAMRA labeled, human	AnaSpec	AS-64519
Fluoromount DAPI	Life Technologies	00-4959-52
Monocyte Isolation Kit II	Miltenyi Biotech	130-091-153
Hygromycin B	Thermo Fisher	10687010
L-Glutamine solution	Sigma	G7513-100ML
SAG	Cayman Chemicals	11914
Dibutyryl cAMP sodium salt	Sigma	D0627-1G
Human Recombinant BDNF	Peprotech	450-02
Human Recombinant NT3	Peprotech	450-03
Legendplex human Microglia/Macrophage Panel	Biolegend	740503
FLICA 660 Caspase 3/7 Kit	BioRad	ICT9125

Primary antibodies used for immunocytochemistry

**Antigen (gene)**	Species	Isotype	Clonality	Manufacturer	Cat.No.	Dilution
IBA1 (*AIF1*)	Rabbit	synthetic	Polyclonal	Wako	019-19741	1:2000
β3-tubulin (*TUBB3*)	Mouse	IgG2a, κ	Monoclonal	Biolegend	801201	1:1000
TAU (*MAPT*)	Rabbit	Recombinant human tau (*E. coli*)	Polyclonal	Dako	A0024	1:100
HLA-DR	Mouse	IgG2a, κ	Monoclonal	Santa Cruz Biotechnology	sc-69673	1:100
TMEM119	Rabbit	IgG	Polyclonal	Abcam	ab185333	1:500
CX3CR1	Mouse	IgG2a, κ	Monoclonal	Santa Cruz Biotechnology	sc-377227	1:50
PU.1 (*SPI1*)	Mouse	IgG1, κ	Monoclonal	eBioscience	14-9819-82	1:200
C/EBPβ (*CEBPB*)	Mouse	IgG2a, κ	Monoclonal	Santa Cruz Biotechnology	sc-7962	1:50
OCT3/4 (*POU5F1*)	Mouse	IgG1, κ	Monoclonal	Santa Cruz Biotechnology	sc-5279	1:50
NANOG	Mouse	IgG1, κ	Monoclonal	Santa Cruz Biotechnology	sc-293121	1:50
KI-67	Rabbit	IgG	Monoclonal	Abcam	Ab16667	1:250
GFAP	Chicken	IgY	Polyclonal	Merck/ Millipore	AB5541	1:500

Secondary antibodies used for immunocytochemistry

Antibody	Species	Isotype	Clonality	Manufacturer	Cat.No.	Dilution
Goat anti-mouse IgG, Alexa Fluor 488	Goat	IgG	Polyclonal	Invitrogen	A-11029	1:500
Goat anti-rabbit, Alexa Fluor 594	Goat	IgG	Polyclonal	Invitrogen	A-11012	1:500
Donkey anti-mouse IgG-Cy2	Donkey	IgG	Polyclonal	dianova	715-225-150	1:200
Donkey anti-rabbit IgG-Cy3	Donkey	IgG	Polyclonal	dianova	711-165-152	1:200
Donkey anti-rabbit IgG-Alexa Fluor 488	Donkey	IgG	Polyclonal	ThermoFisher	A21206	1:1000
Donkey anti-mouse IgG-Alexa Fluor 568	Donkey	IgG	Polyclonal	ThermoFisher	A10037	1:1000
Donkey anti-chicken IgY (IgG)	Donkey	IgY	Polyclonal	Jackson ImmunoResea.	703-606-155	1:1000

Primary antibodies used for flow cytometry

**Antibodies Flow Cytometry**	Species	Isotype	Clonality	Fluorophore	Manufacturer	Cat. No.	Dilution
CD45	Mouse	IgG1, κ	Monoclonal	FITC	Biolegend	368508	1:100
CD11b	Rat	IgG2b, κ	Monoclonal	Pacific Blue	Biolegend	101224	1:100
CD11c	Mouse	IgG1, κ	Monoclonal	AlexaFluor 700	Biolegend	337220	1:100
CX3CR1	Rat	IgG2b, κ	Monoclonal	APC	Biolegend	341610	1:100
TREM2	Rat	IgG2b, κ	Monoclonal	APC	R&D Systems	FAB17291A	1:100
P2RY12	Mouse	IgG2a, κ	Monoclonal	PE	Biolegend	392104	1:100
CD14	Mouse	IgG2a, κ	Monoclonal	PE	Biolegend	301806	1:100
CD115 (CSF-1R)	Rat	IgG1, κ	Monoclonal	PE	Biolegend	347304	1:50
CD16	Mouse	IgG1, κ	Monoclonal	FITC	Biolegend	360716	1:50
ENTPD (CD39)	mouse	IgG1, κ	Monoclonal	FITC	eBioscience	11-0399-73	1:50

Primers used for quantitative real-time PCR (qPCR)

**Gene**	**Orientation**	**Primer sequence**
GAPDH	Fwd	GTGAAGGTCGGAGTCAACGG
	Rev	TGAAGGGGTCATTGATGGCA
OCT4	Fwd	GTGGAGGAAGCTGACAACAA
	Rev	ATTCTCCAGGTTGCCTCTCA
NANOG	Fwd	AGCAGATGCAAGAACTCTCCAA
	Rev	TGAGGCCTTCTGCGTCACAC
SPI1 (total)	Fwd	CCCCCTCCATCAGAAGACCT
	Rev	AAGTCCCAGTAATGGTCGCT
SPI1 (endogenous)	Fwd	CCTGCAATGTCAAGGGAGGG
	Rev	CCAATGCAGAGCCCCTCAG
CEBPB (total)	Fwd	GACGAGTACAAGATCCGGCG
	Rev	ACAGCTGCTCCACCTTCTTC
CEBPB (endogenous)	Fwd	GGGAGCCCGTCGGTAATTTT
	Rev	CATGTGCGGTTGGTTTGGAC
RUNX1	Fwd	GCCTTCAGAAGAGGGTGCAT
	Rev	CTGGCATCGTGGACGTCTCTA
IRF8	Fwd	TCGACACCAGCCAGTTCTTC
	Rev	GCTGCTCAATCTGCACGAGA
P2RY12	Fwd	CCAGGGTCAGATTACAAGAGCA
	Rev	CTGCAGAGTGGCATCTGGTAT
GPR34	Fwd	GCGACTATTACCAAATAGAAGACAA
	Rev	GCTGAGAAGTTTTGTGGCGG
MERTK	Fwd	CTTCTCCATGGCCACAGGTT
	Rev	ATACTGAAAAGGTGGGGCGG
C1QA	Fwd	GAGCACCAGACGGGAAGAAA
	Rev	TAAGGCCTTGGATGCCTGTC

PROS1	Fwd	TTCCCGTCTCAGAGGCAAAC
	Rev	TGTTGCTTTGACAAAAAGCATATAG
GAS6	Fwd	CGACCCCGAGACGGATTATT
	Rev	CTTCCTATCGCAGGGGTTGG
HEXB	Fwd	GGGAGCATTACGAGGTTTAGAG
	Rev	GGTGGATTCATTGATGGTGAAAG
CD14	Fwd	TGCCGCTGTGTAGGAAAGAA
	Rev	CGCGCTCCATGGTCGATA
TREM2	Fwd	ATGGCAGTGAGGCTGACAC
	Rev	GAGGCTCCTGGAGATGCTGTG
CX3CR1	Fwd	CCCCTGGAGGCGTTTAAGTT
	Rev	ACAGGCCTCAGCCAAATCAT
MYB	Fwd	GCCAATTATCTCCCGAATCGA
	Rev	ACCAACGTTTCGGACCGT
CSF1R	Fwd	CTCTGCAGGAGCCCACAC
	Rev	TTCCAGCGGACCTGGTACT
TMEM119	Fwd	AGCACGGACTCTCTCTTCCAG
	Rev	GTGCCCCCAGGACCAGTTC
AIF1	Fwd	ACCAGGGATTTACAGGGAGGA
	Rev	TGGAGGGCAGATCCTCATCA
PTPRC	Fwd	GGCTTTGCCTTTCTGGACAC
	Rev	TTGCTGTAGTCAATCCAGTGGG
CD68	Fwd	GCTACTGGCAGCCCAGG
	Rev	CGTGAAGGATGGCAGCAAAG
HLA-DRA	Fwd	ATGGCCATAAGTGGAGTCCC
	Rev	TCGCCTGATTGGTCAGGATTC
SALL1	Fwd	CATTTCCAATCCGACCCCGA
	Rev	CAAAGAACTCGGCACAGCAC
MAFB	Fwd	ACTTTGTCTTGGGGCACACT
	Rev	GGGACCTCTCGGTTCTCTCT
MEF2C	Fwd	AGTGCAGGTAACACAGGTGG
	Rev	AGCAGACCTGGTGAGTTTCG
JUN	Fwd	GAGCTGGAGCGCCTGATAAT
	Rev	CCCTCCTGCTCATCTGTCAC
FOS	Fwd	GGAGAATCCGAAGGGAAAGGA
	Rev	GTTGGTCTGTCTCCGCTTGG

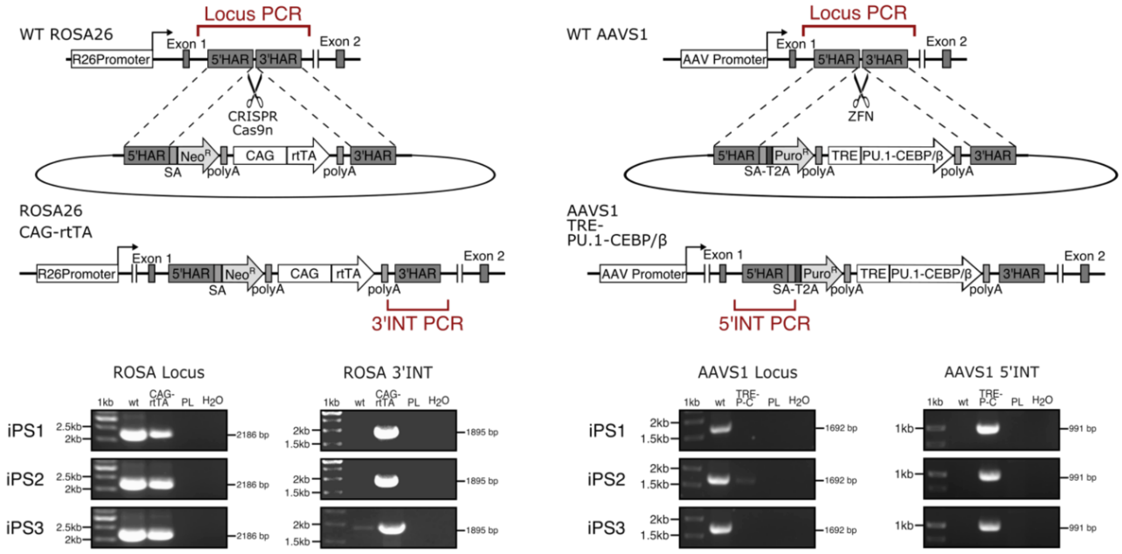

Fig. S1. (Related to Figure 1): Genotyping strategy to verify correct donor cassette insertion into the genomic safe harbors hROSA26 and AAVS1.

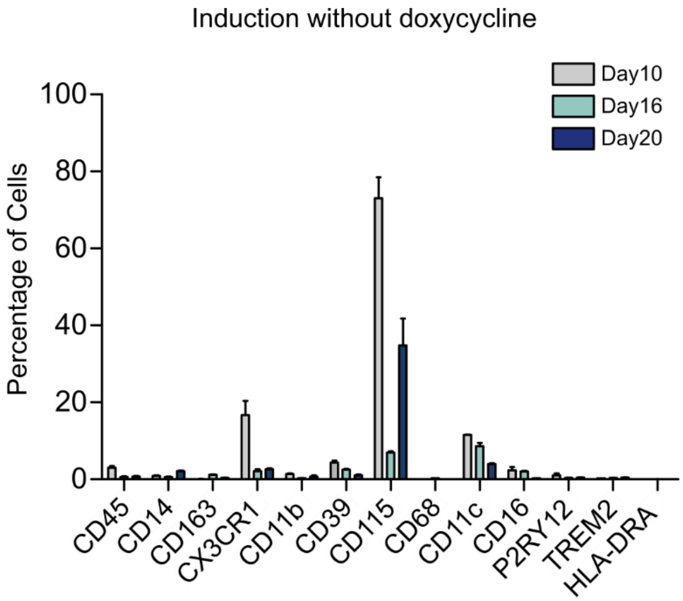

Fig. S2. (Related to Figure 2): Flow cytometry of cells cultivated in identical standard medium conditions described in this protocol, without the addition of doxycycline and therefore without overexpression of transgenic PU.1 and C/EBPβ.

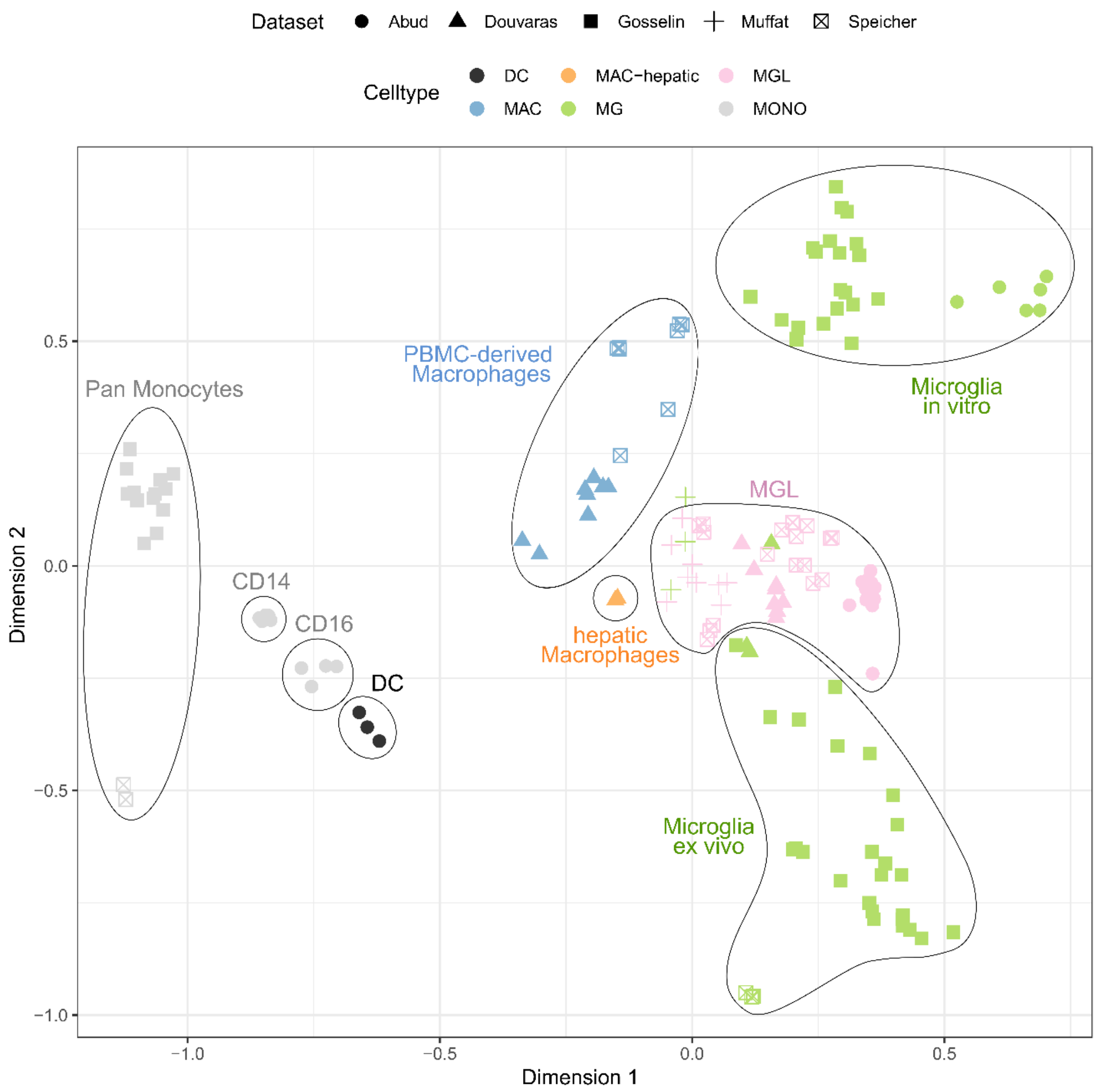

Fig. S3. (Related to Figure 3): MDS plot showing cell clusters after exclusion of progenitor cells (hiPSCs, HPCs, NPCs) and cortical cells from the analysis.

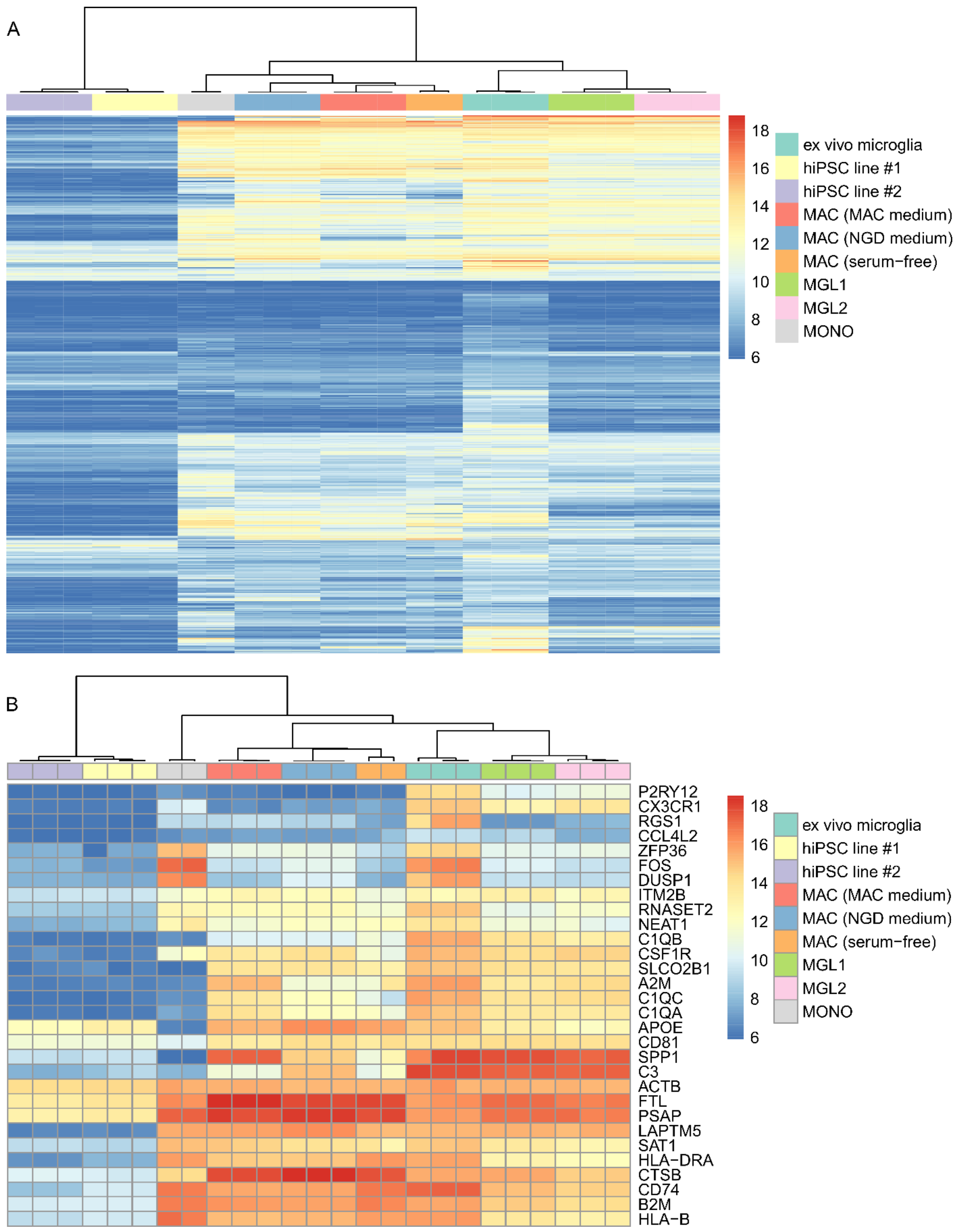

Fig. S4. (Related to Figure 3): Heatmaps showing expression of genes enriched in primary human microglia *ex vivo* as defined by Gosselin et al. (18). (A) Table S2 from (18) (B) Top-30 of Table S2 from (18) in human *ex vivo* microglia vs. MGLs, macrophages, monocytes and hiPSCs.

A

	D0	D2	D4	D6 – D8	D10 – D18
Condition 1:	Basal medium + FGF2 + BMP4 + CHIR + VEGF-A + LY + Activin A	Basal medium + FGF2 + SCF + IL-3 + IL-6 + M-CSF	Basal medium + FGF2 + SCF + IL-3 + IL-6 + M-CSF	Basal medium + M-CSF	NGD complete
Condition 2:	Basal medium + FGF2 + BMP4 + CHIR + VEGF-A + LY + Activin A	Basal medium + M-CSF + FGF2	Basal medium + M-CSF + FGF2	Basal medium + M-CSF	NGD complete
Condition 3:	Basal medium + FGF2 + BMP4 + LY + Activin A	Basal medium + FGF2 + SCF + IL-3 + IL-6 + M-CSF	Basal medium + FGF2 + SCF + IL-3 + IL-6 + M-CSF	Basal medium + M-CSF	NGD complete
Condition 4:	Basal medium + FGF2 + BMP4 + LY + Activin A	Basal medium + M-CSF + FGF2	Basal medium + M-CSF + FGF2	Basal medium + M-CSF	NGD complete
Condition 5:	Basal medium + FGF2 + LY + Activin A	Basal medium + M-CSF + FGF2	Basal medium + M-CSF + FGF2	Basal medium + M-CSF	NGD complete
Condition 6:	75% iPS medium + 25% NGD complete	50% iPS medium + 50% NGD complete	25% iPS medium + 75% NGD complete	NGD complete	NGD complete
Condition 7:	Basal medium + FGF2 + BMP4 + VEGF-A + LY + Activin A + WNT-C59 (10nM)	Basal medium + FGF2 + SCF + IL-3 + IL-6 + M-CSF	Basal medium + FGF2 + SCF + IL-3 + IL-6 + M-CSF	Basal medium + M-CSF	NGD complete
Condition 8:	Basal medium + FGF2 + BMP4 + VEGF-A + LY + Activin A + WNT-C59 (10nM)	Basal medium + M-CSF + FGF2	Basal medium + M-CSF + FGF2	Basal medium + M-CSF	NGD complete

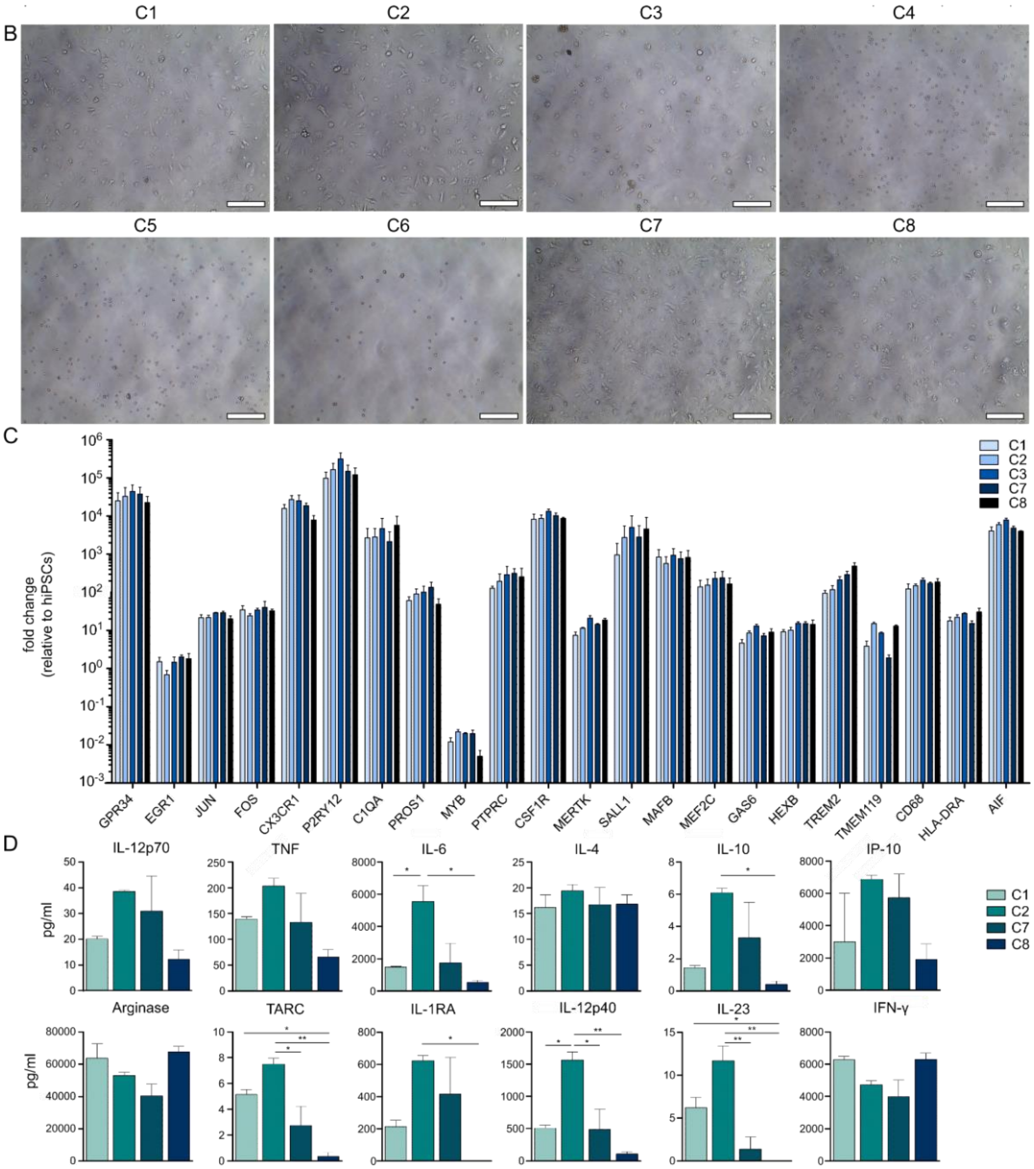

Fig. S5. (Related to Figures 2, 3, and 4): Growth factor reduction experiments. (A) Media composition of 8 different media conditions. C1 represents the standard protocol presented within this publication. (B) Bright field images of D18 MGLs (scale = 100 µm). (C) Gene expression analysis of D18 MGLs of all conditions containing viable cells. (D) Cytokine secretion assay comparing C1, 2, 7 and 8 after 48h stimulation with LPS/IFN-γ. Statistical significance was determined using Bonferroni-corrected one-way ANOVA (* $p < 0,05$; ** $p < 0,01$).

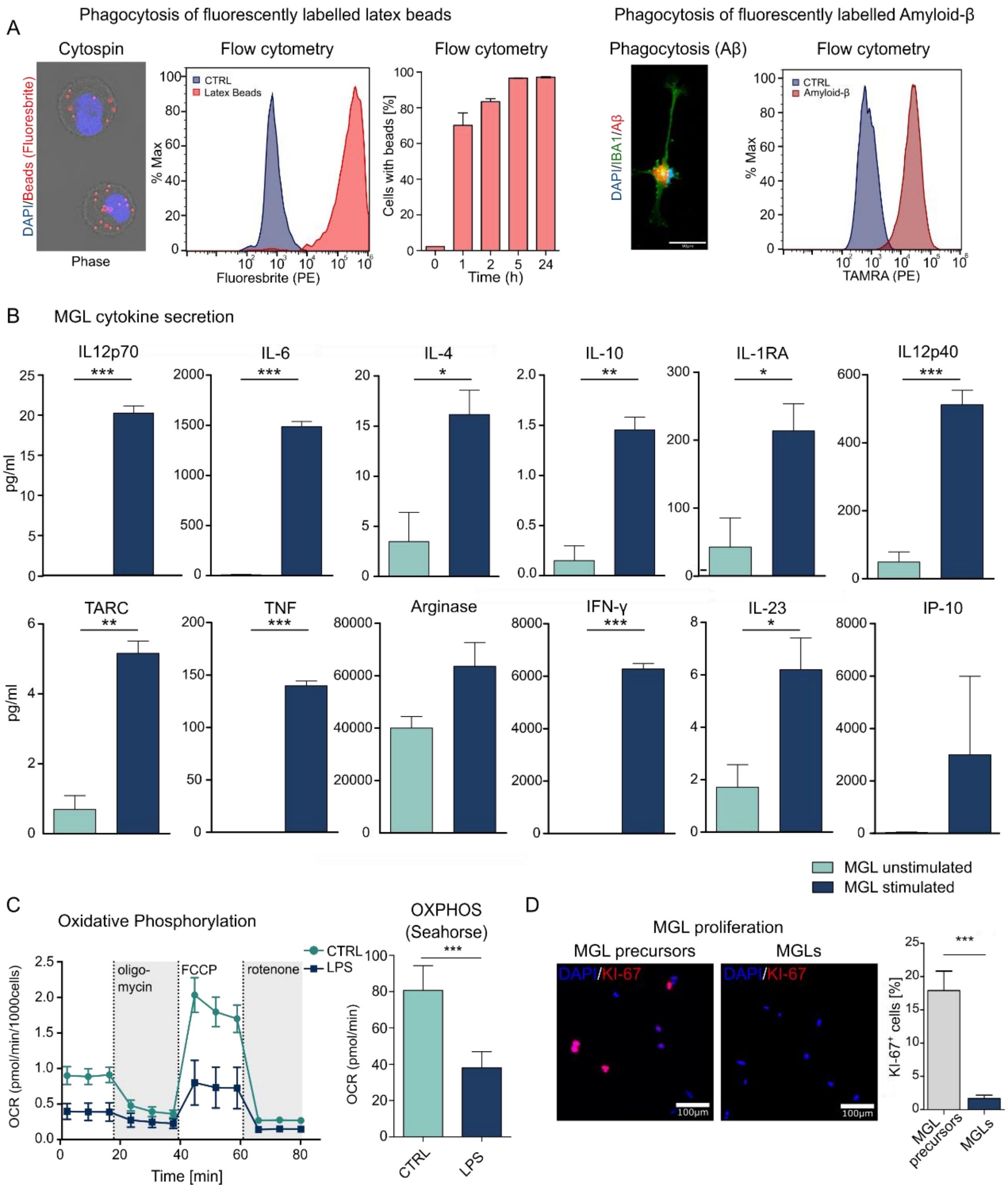

Fig. S6. (Related to Figure 4): (A) Phagocytosis assay using fluorescently labeled latex beads analyzed by cytospin and flow cytometry (left). Phagocytosis assay using labeled Amyloid-β analyzed by ICC and flow cytometry (right). (B) Cytokine secretion assay comparing MGLs in an unstimulated condition and stimulated with LPS/IFN-γ for 48h. Significance was determined with an unpaired t-test (* p < 0,05; ** p < 0,01; *** p < 0,001). (C) Oxygen consumption rate (OCR) of MGLs cultivated under control conditions and after LPS stimulation (left). OCR quantification at baseline under both conditions (right). (D) KI-67 staining in MGL precursor cells and MGLs and quantification of KI-67 positive cells in both groups. Statistical significance was determined using Bonferroni-corrected one-way ANOVA (*** p < 0,001).

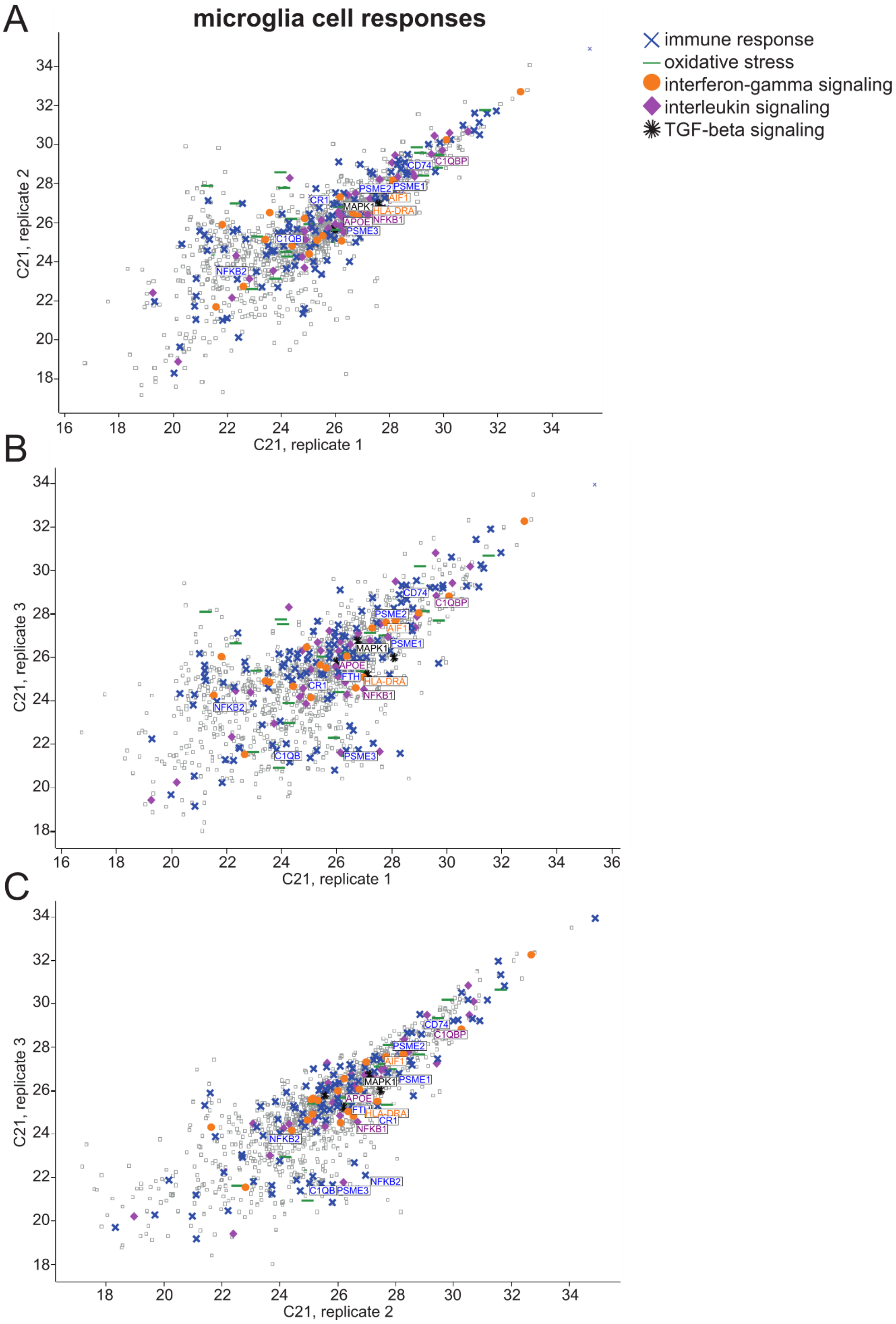

Fig. S7A. (Related to Figure 4): Correlation of proteome data set with proteins linked to microglia cell responses. Microglia cell responses: All mapped proteins were searched by their GO-BP (GO-biological processes) terms for proteins associated with immune response (symbol: blue, diagonal cross), oxidative stress (symbol: green, horizontal dash), interferon-gamma signalling (symbol: orange, filled circle) and interleukin signalling (symbol: magenta, filled diamond) or the TGF-beta pathway (symbol: black, star).

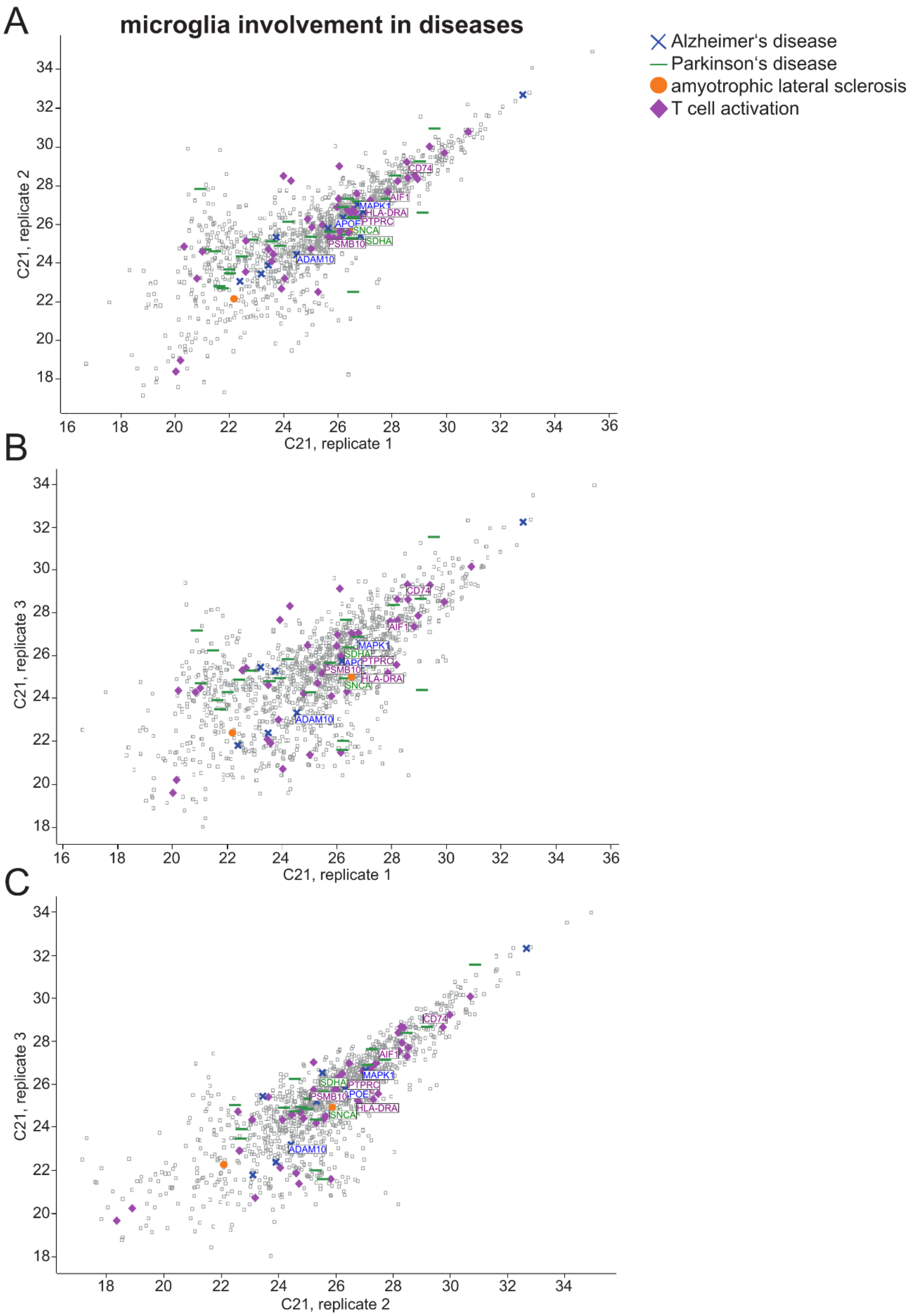

Fig. S7B. (Related to Figure 4): Correlation of proteome data set with proteins linked to microglia involvement in diseases: Proteins related to neurological disorders like Alzheimer’s disease (symbol: blue, diagonal cross), Parkinson’s disease (symbol: green, horizontal dash), amyotrophic lateral sclerosis (symbol: orange, filled circle) and T cell activation in general (symbol: magenta, filled diamond) are marked. Labelling of the matched proteins was performed based on their corresponding gene symbols.

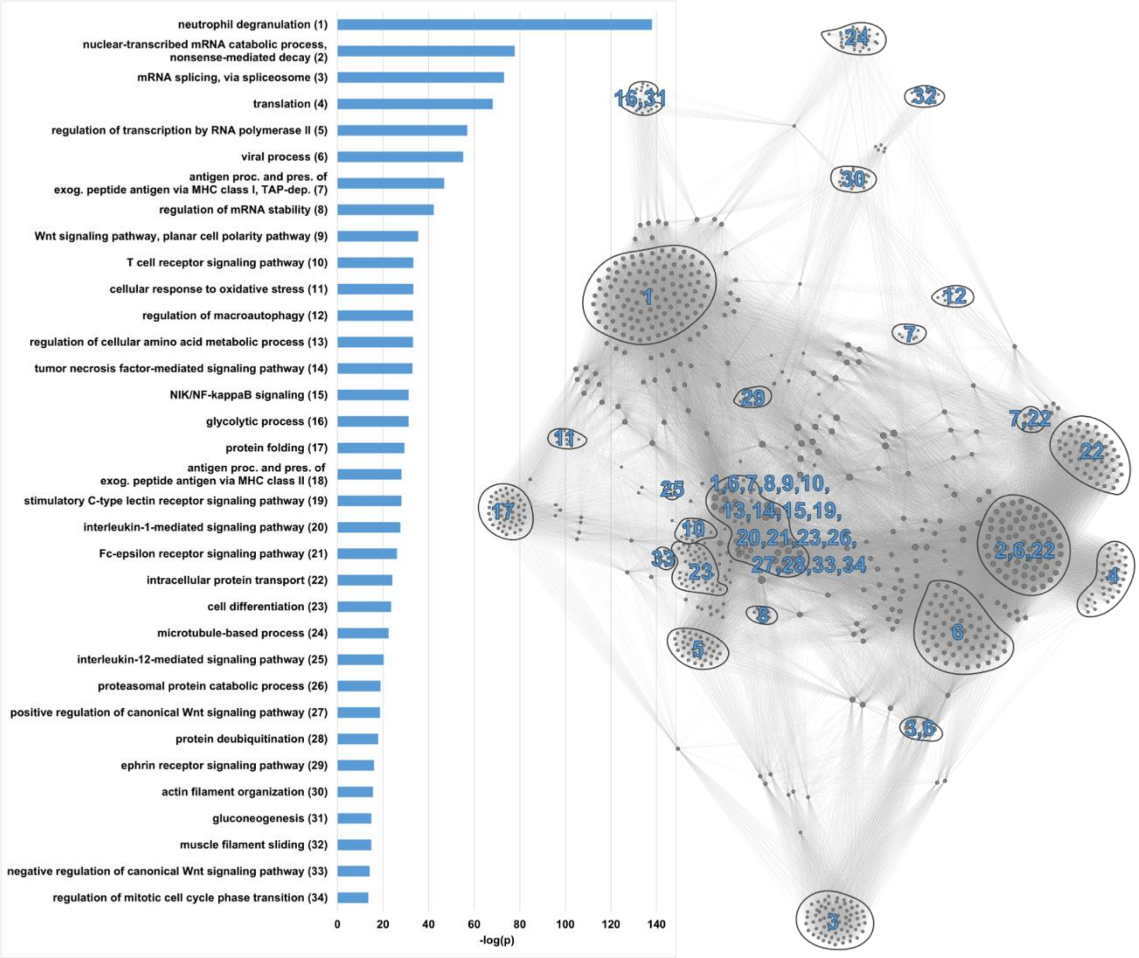

Fig. S8. (Related to Figure 4): Force-directed graph showing shared GO terms of the proteome, that are depicted as the connections (edges) in the graph. Dense protein clusters are highly similar whereas dissimilar proteins show a spatial distance.

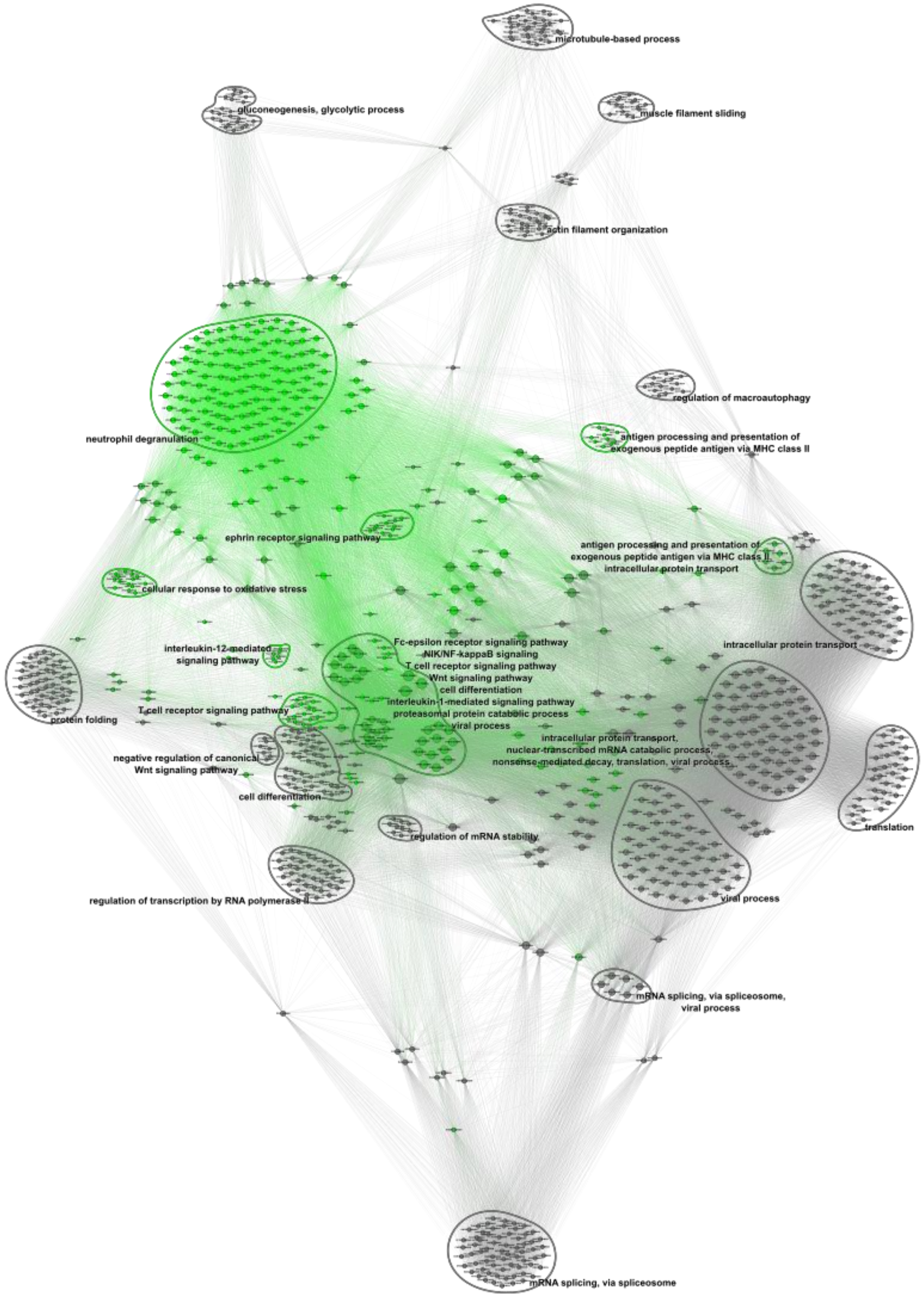

Fig. S9. (Related to Figure 4): Selection of microglia-relevant terms coded by color grading from green to gray. This figure includes the protein IDs for a detailed inspection.

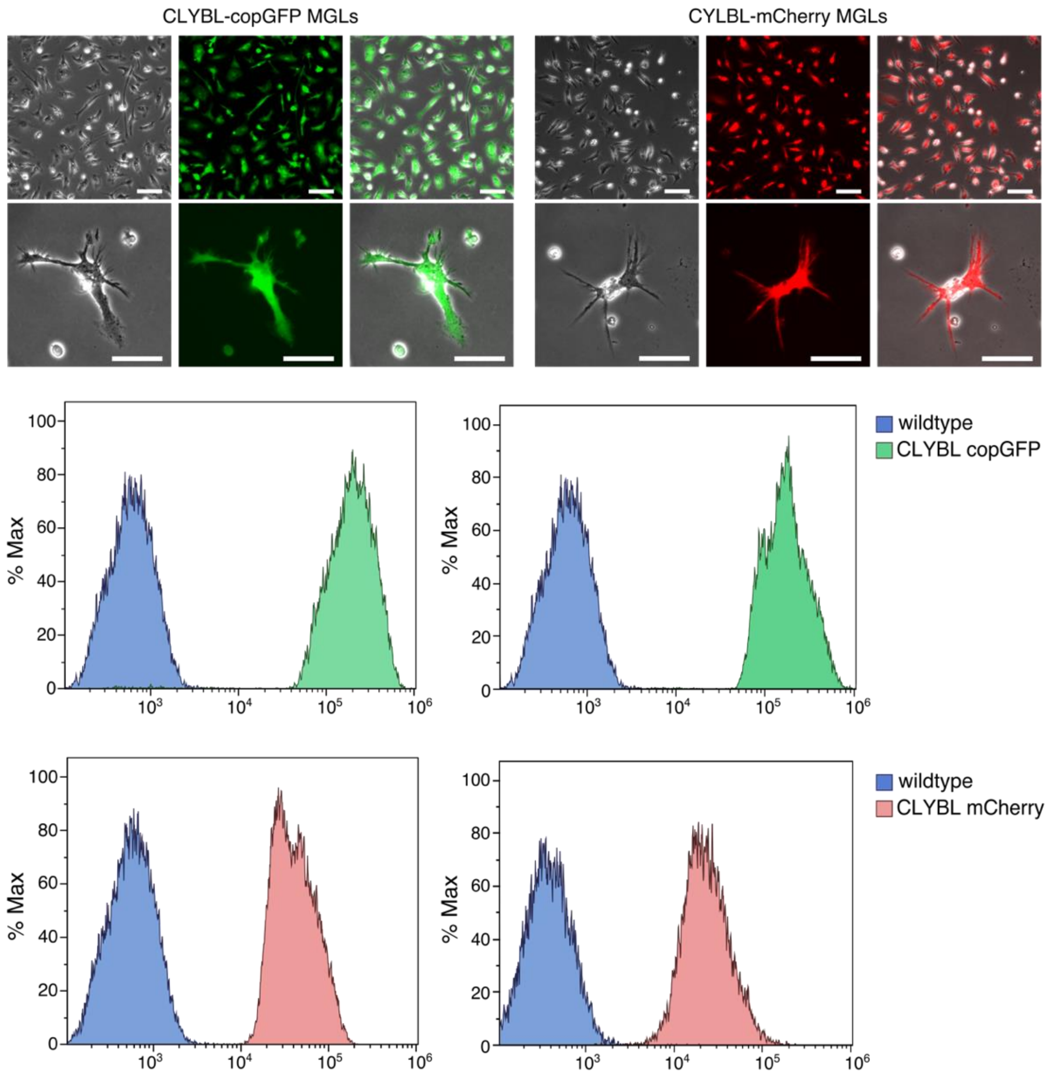

Fig. S10. (Related to Figure 5): MGL reporter cell lines that were generated by targeting the *CLYBL* locus with copGFP or mCherry. Reporter cell lines were used for coculture experiments.

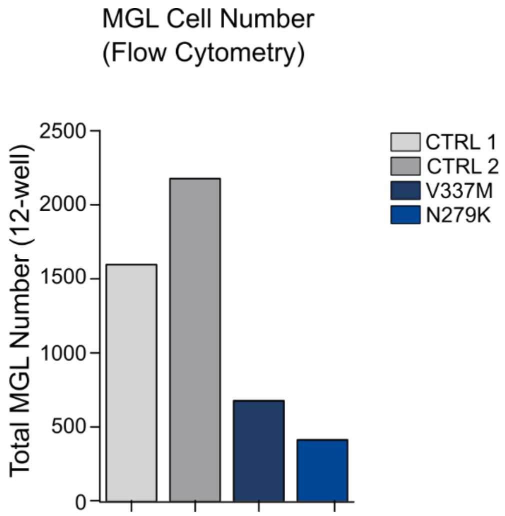

Fig. S11. (Related to Figure 5): Determination of microglia cell numbers in coculture with wild-type neurons and neurons carrying different *MAPT* mutations, using flow cytometry.

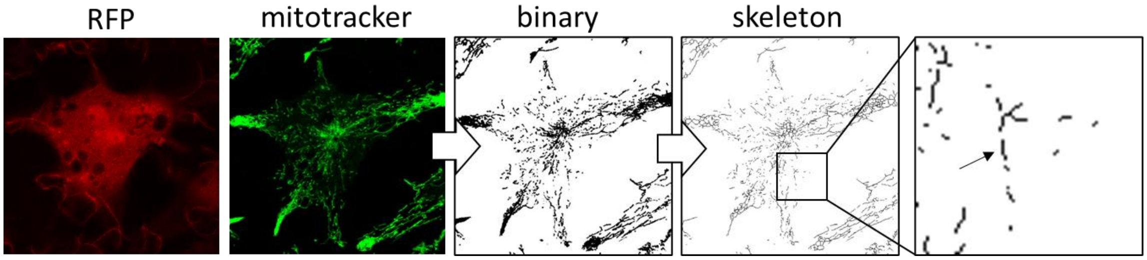

Fig. S12. (Related to Figure 5): Representative confocal images of MGL in coculture with neurons derived from a reporter cell line (expressing mCherry, see Fig. S7) stained with mitotracker and subsequent processing for mitochondrial morphology analysis. Arrow indicates representative single mitochondria skeleton that was incorporated into analysis of the average branch length.

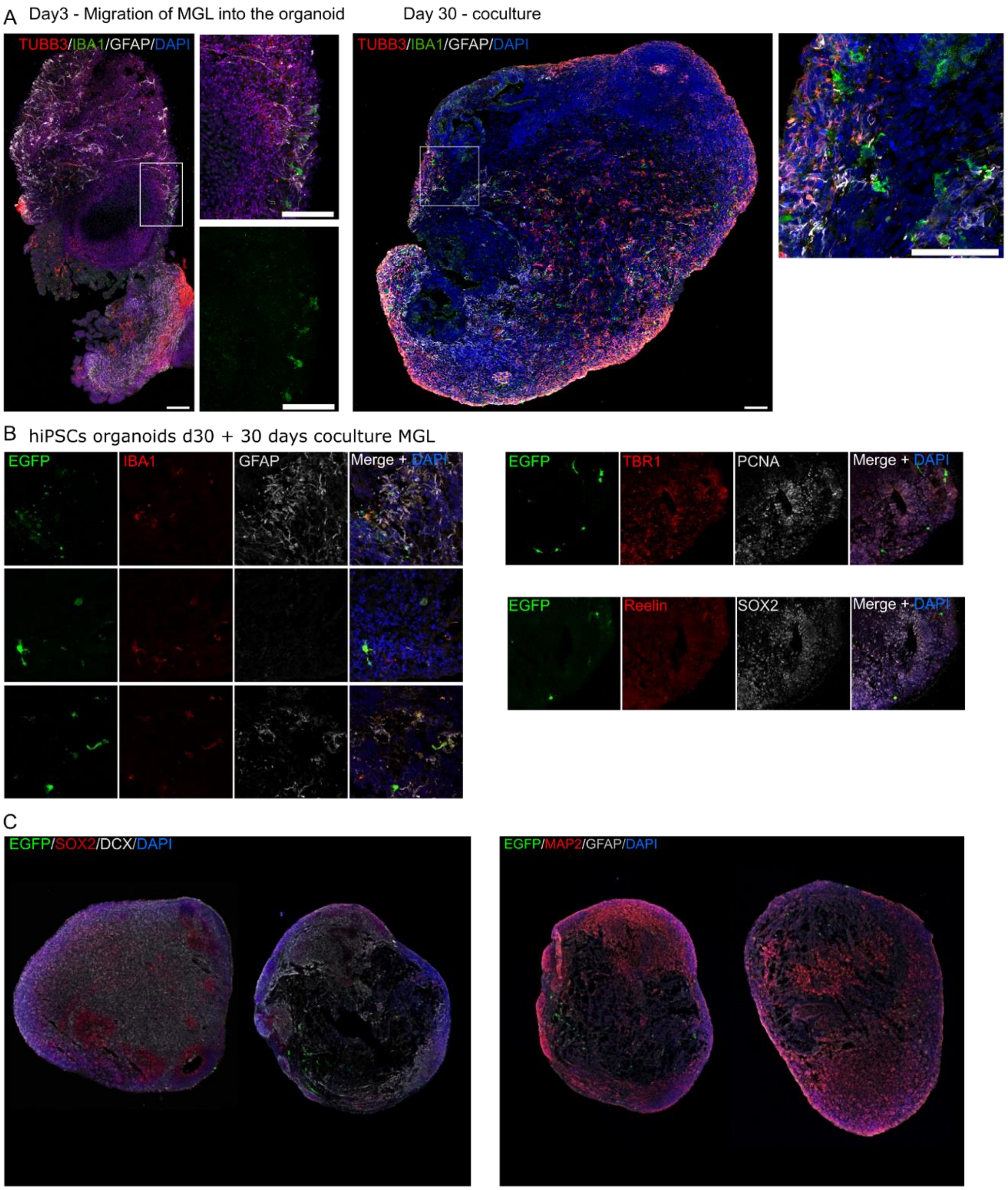

Fig. S13. (Related to Figure 6): (A) Immunohistochemistry (IHC) for the pan-neuronal marker β3-tubulin (TUBB3; red), the microglia marker IBA1 (green) and the astrocyte marker GFAP (white) in two representative cortical organoids containing microglia after 30 days of coculture. (Scale bar = 100 µm). (B-C) Organoid organization with integrated reporter MGLs (expressing EGFP) at day 60.

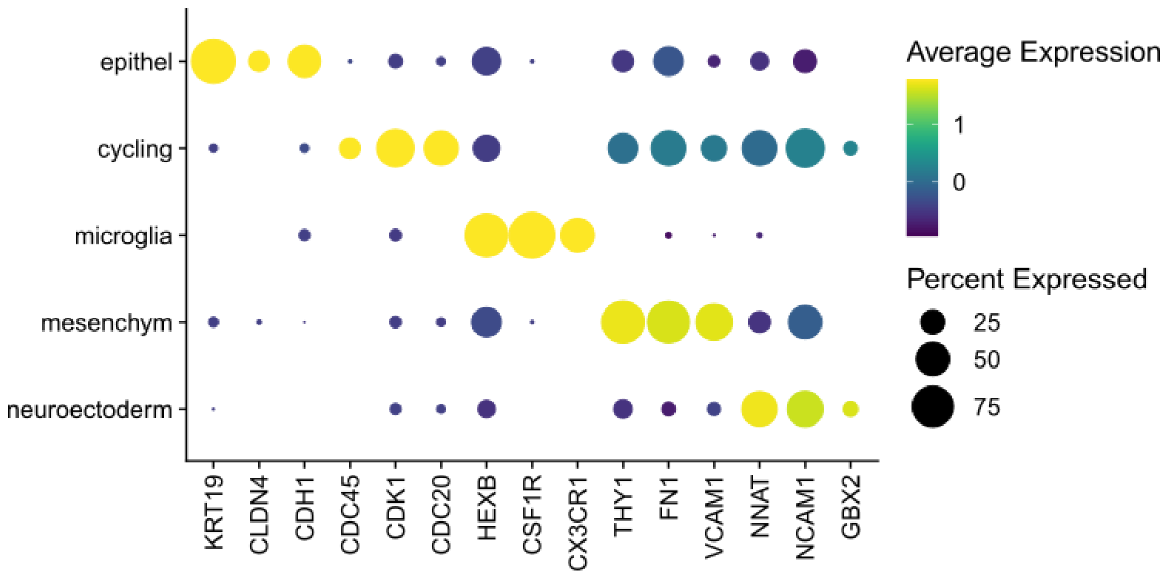

Fig. S14. (Related to Figure 6): Marker genes of main cell clusters in Fig. 6*A-B* are depicted. Colors encode the average gene expression and dot size represents the percentage of cells expressing the respective gene paste legend here.

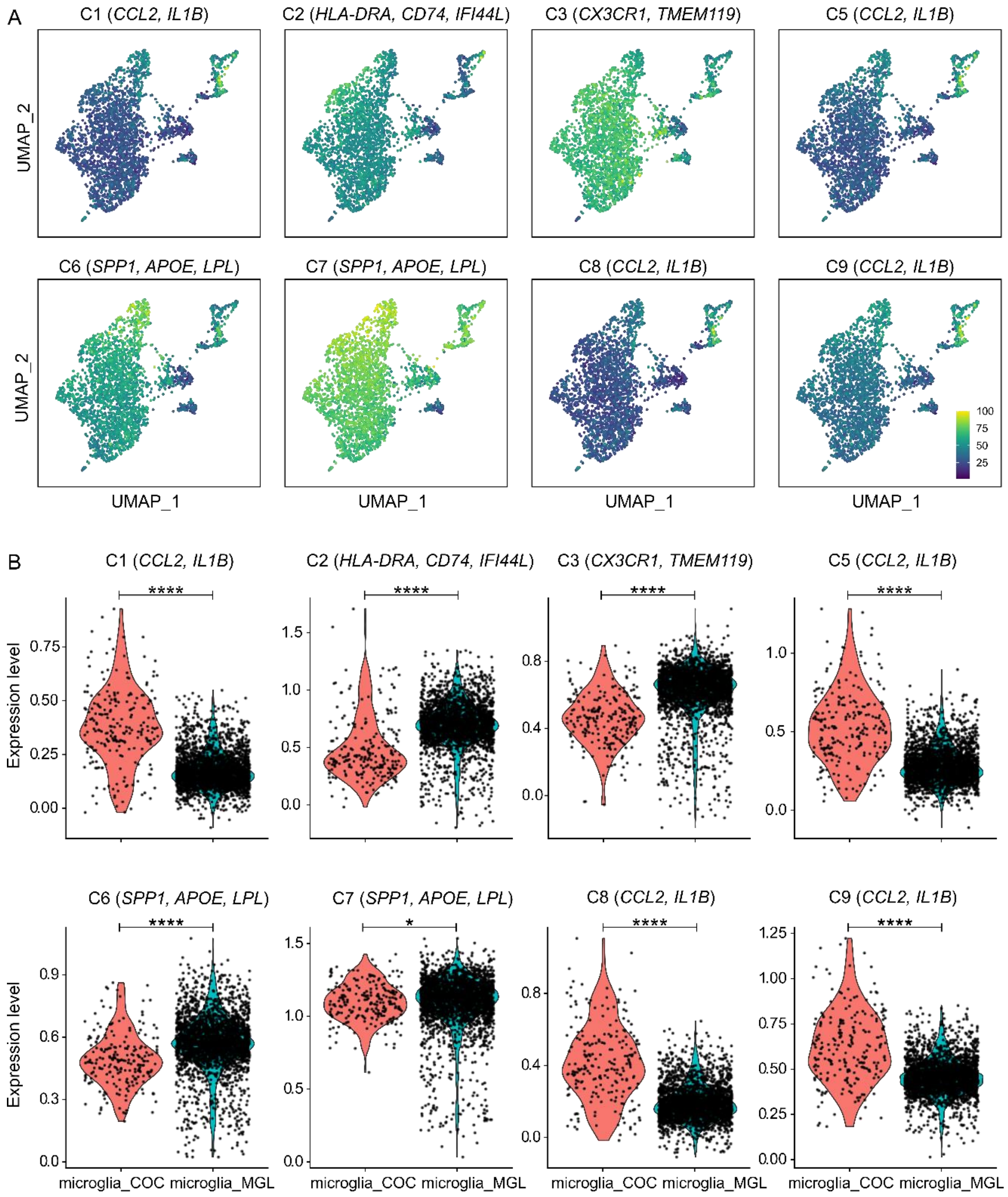

Fig. S15. (Related to Figure 6): (A) Feature plots depicting gene set scores of the different microglia clusters based on previously defined clusters of primary human microglia (Sankowski et al. (21)). (B) Violin plots displaying the gene set scores of microglia cocultured with organoids (microglia_COC, red) and microglia from the monoculture (microglia_MGL, blue) with a gene set from human microglia (Sankowski et al., 2019 (21)). P-values were computed using the Wilcoxon rank sum test (*p < 0,05; **** p ≤ 0,0001).

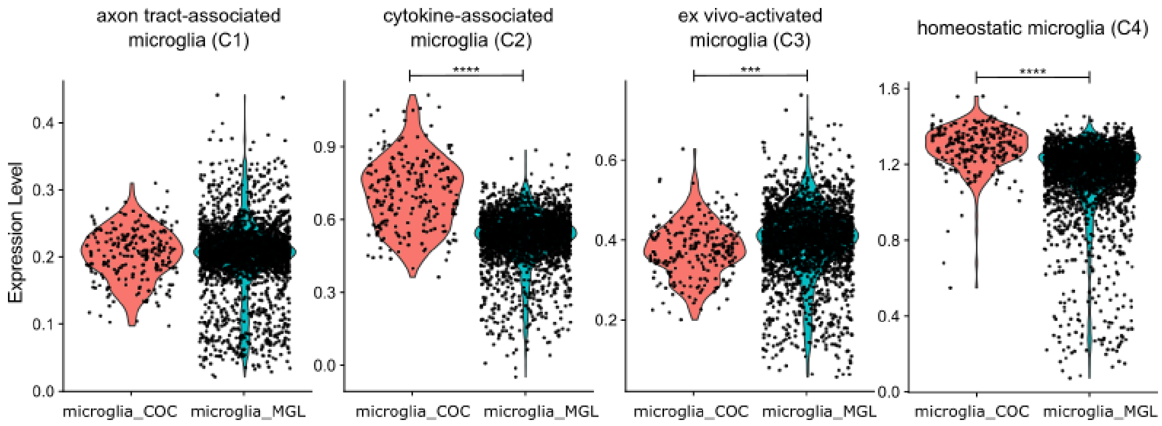

Fig. S16. (Related to Figure 6): Violin plots displaying the gene set scores of microglia cocultured with organoids (microglia_COC, red) and microglia from the monoculture (microglia_MGL, blue) with a gene set from human microglia (Popova et al., 2022 (22)). P-values were computed using the Wilcoxon rank sum test (***p < 0,001; **** p ≤ 0,0001).

Dataset S1. Marker genes of clusters in the organoid-microglia coculture (corresponding to Fig. 6*A*) *avg_log₂FC:* $\log_2$ fold change of the average expression between the cluster vs. all remaining clusters; *p_val*: p value; *p_val_adj:* adjusted p values (based on Bonferroni correction). *pct.1:* percentage of cells with the gene detected in the cluster; *pct.2:* percentage of cells with the gene detected in all remaining clusters.

Dataset S2. Marker genes in microglia in coculture with organoids and MGLs *in vitro* (corresponding to Fig. 6*C*).
avg_log$_2$FC: $\log_2$ fold change of the average expression between the cluster vs. all remaining clusters; *p_val*: p value; *p_val_adj:* adjusted p values (based on Bonferroni correction). *pct.1:* percentage of cells with the gene detected in the cluster; *pct.2:* percentage of cells with the gene detected in all remaining clusters.

Dataset S3. Differentially expressed genes of MGLs *in vitro* and MGLs in organoids versus previously defined clusters of primary human microglia of Sankowski et al. (21); (corresponding to Fig. 6*E* and 6*G* and S14)

Dataset S4. Differentially expressed genes of MGLs *in vitro* and MGLs in organoids versus previously defined microglia clusters of Popova et al. (22) (corresponding to Fig. 6*H*)
avg_log$_2$FC: $\log_2$ fold change of the average expression between the cluster vs. all remaining clusters; *p_val*: p value; *p_val_adj:* adjusted p values (based on Bonferroni correction). *pct.1:* percentage of cells with the gene detected in the cluster; *pct.2:* percentage of cells with the gene detected in all remaining clusters.

Dataset S5. Marker genes of MGL subclusters (corresponding to Fig. 6*D-E*).

avg_log$_2$FC: $\log_2$ fold change of the average expression between the cluster vs. all remaining clusters; *p_val*: p value; *p_val_adj:* adjusted p values (based on Bonferroni correction). *pct.1:* percentage of cells with the gene detected in the cluster; *pct.2:* percentage of cells with the gene detected in all remaining clusters.

Dataset S6. Marker genes for cell-communication between microglia and neuroectodermal cells generated using CellPhoneDB (corresponding to Fig. 6*A*).

avg_log$_2$FC: $\log_2$ fold change of the average expression between the cluster vs. all remaining clusters; *p_val*: p value; *p_val_adj:* adjusted p values (based on Bonferroni correction). *pct.1:* percentage of cells with the gene detected in the cluster; *pct.2:* percentage of cells with the gene detected in all remaining clusters.

Dataset S7. Gene ontology analysis based on Dataset S6.

SI References

1. H. Kilpinen, *et al.*, Common genetic variation drives molecular heterogeneity in human iPSCs. *Nature* **546**, 370–375 (2017).
2. K.-P. Kim, *et al.*, Reprogramming competence of OCT factors is determined by transactivation domains. *Sci. Adv.* **6**, 1–17 (2020).
3. M. R. Mizee, *et al.*, Isolation of primary microglia from the human post-mortem brain: effects of ante- and post-mortem variables. *Acta Neuropathol. Commun.* **5**, 16 (2017).
4. A. Bertero, *et al.*, Optimized inducible shRNA and CRISPR/Cas9 platforms for in vitro studies of human development using hPSCs. *Development* **143**, 4405–4418 (2016).
5. M. Pawlowski, *et al.*, Inducible and Deterministic Forward Programming of Human Pluripotent Stem Cells into Neurons, Skeletal Myocytes, and Oligodendrocytes. *Stem cell reports* **8**, 803–812 (2017).
6. T. Cerbini, *et al.*, Transcription Activator-Like Effector Nuclease (TALEN)-Mediated CLYBL Targeting Enables Enhanced Transgene Expression and One-Step Generation of Dual Reporter Human Induced Pluripotent Stem Cell (iPSC) and Neural Stem Cell (NSC) Lines. *PLoS One* **10**, e0116032 (2015).
7. J. Muffat, *et al.*, Efficient derivation of microglia-like cells from human pluripotent stem cells. *Nat. Med.* **22**, 1358–1367 (2016).
8. M. Ehrlich, *et al.*, Distinct Neurodegenerative Changes in an Induced Pluripotent Stem Cell Model of Frontotemporal Dementia Linked to Mutant TAU Protein. *Stem cell reports* **5**, 83–96 (2015).
9. A.-L. Hallmann, *et al.*, Astrocyte pathology in a human neural stem cell model of frontotemporal dementia caused by mutant TAU protein. *Sci. Rep.* **7**, 42991 (2017).
10. A. J. Valente, L. A. Maddalena, E. L. Robb, F. Moradi, J. A. Stuart, A simple ImageJ macro tool for analyzing mitochondrial network morphology in mammalian cell culture. *Acta Histochem.* **119**, 315–326 (2017).
11. M. A. Lancaster, *et al.*, Cerebral organoids model human brain development and microcephaly. *Nature* **501**, 373–9 (2013).
12. M. Ashburner, *et al.*, Gene ontology: tool for the unification of biology. The Gene Ontology Consortium. *Nat. Genet.* **25**, 25–9 (2000).
13. Y. Benjamini, Y. Hochberg, Controlling the False Discovery Rate: A Practical and Powerful Approach to Multiple TestinG. *J. R. Stat. Soc. Ser. B* **57**, 289–300 (1995).
14. C. B. Schroeter, *et al.*, Protein half-life determines expression of proteostatic networks in podocyte differentiation. *FASEB J.* **32**, 4696–4713 (2018).
15. P. Kohli, *et al.*, Label-free quantitative proteomic analysis of the YAP/TAZ interactome. *Am. J. Physiol. Cell Physiol.* **306**, C805-18 (2014).
16. E. M. Abud, *et al.*, iPSC-Derived Human Microglia-like Cells to Study Neurological Diseases. *Neuron* **94**, 278-293.e9 (2017).
17. P. Douvaras, *et al.*, Directed Differentiation of Human Pluripotent Stem Cells to Microglia. *Stem cell reports* **8**, 1516–1524 (2017).
18. D. Gosselin, *et al.*, An environment-dependent transcriptional network specifies human microglia identity. *Science* **356**, 2150 (2017).
19. Y. Hao, *et al.*, Integrated analysis of multimodal single-cell data. *Cell* **184**, 3573-3587.e29 (2021).
20. M. Efremova, M. Vento-Tormo, S. A. Teichmann, R. Vento-Tormo, CellPhoneDB: inferring cell-cell communication from combined expression of multi-subunit ligand-receptor complexes. *Nat. Protoc.* **15**, 1484–1506 (2020).
21. R. Sankowski, *et al.*, Mapping microglia states in the human brain through the integration of high-dimensional techniques. *Nat. Neurosci.* **22**, 2098–2110 (2019).
22. G. Popova, *et al.*, Human microglia states are conserved across experimental models and regulate neural stem cell responses in chimeric organoids. *Cell Stem Cell* **28**, 2153-2166.e6 (2021).
